# Evaluating Ecosystem Characteristics and Ecological Carrying Capacity for Marine Fauna Stock Enhancement Within a Marine Ranching System

**DOI:** 10.3390/ani15020165

**Published:** 2025-01-10

**Authors:** Jie Feng, Haolin Yu, Lingjuan Wu, Chao Yuan, Xiaolong Zhao, Huiying Sun, Cheng Cheng, Yifei Li, Jingyi Sun, Yan Li, Xiaolong Wang, Yongjun Shang, Jiangling Xu, Tao Zhang

**Affiliations:** 1 North China Sea Marine Forecasting Center of State Oceanic Administration, Qingdao 266061, China; f823725486@163.com (J.F.); vivioceangk@163.com (L.W.); yuan-chao-hi@163.com (C.Y.); zhaoxiaolong@ncs.mnr.gov.cn (X.Z.); shy0517@126.com (H.S.); hdliyifei@163.com (Y.L.); candybutterfly@126.com (J.S.); 2 Key Laboratory of Ecological Prewarning, Protection and Restoration of Bohai Sea, Ministry of Natural Resources, Qingdao 266033, China; 3 Shandong Provincial Key Laboratory of Marine Ecological Environment and Disaster Prevention and Mitigation, Qingdao 266061, China; 4 Laboratory for Marine Science and Technology, Qingdao National Laboratory for Marine Science and Technology, Qingdao 266237, China; yuhaolin777@outlook.com (H.Y.); jsrgsha@163.com (C.C.); shangyongjun@stu.ouc.edu.cn (Y.S.); 5 Center for Ocean Mega-Science, Chinese Academy of Sciences, Qingdao 266400, China; 6 CAS Engineering Laboratory for Marine Ranching, Institute of Oceanology, Chinese Academy of Sciences, Qingdao 266400, China; 7 CAS Key Laboratory of Marine Ecology and Environmental Sciences, Institute of Oceanology, Chinese Academy of Sciences, Qingdao 266400, China; 8 Guangxi Academy of Oceanography, Nanning 530022, China; 2005304001@163.com; 9 Marine Science Research Institute of Shandong Province, Qingdao 266104, China; wxl307@163.com

**Keywords:** marine ranching, stock enhancement, index system, carrying capacity, scenarios simulation

## Abstract

China has recently launched extensive marine ranching projects, highlighting the need for scientific evaluation of ecosystem structure and function to guide their development. This study established two energy flow models and an evaluation index system to assess the structure, function, carrying capacity, and ecological status of both a marine ranching ecosystem and a nearby control site in the Beibu Gulf. The results show that the ranching ecosystem outperformed the control ecosystem in terms of food chain length, system size, and ecological carrying capacity of economically important species. The ranching ecosystem was classified as “relatively good”, while the control ecosystem was deemed “relatively poor”, which may confirm the success of the ranching efforts. Mussels, large crabs, and scorpaenidae were identified as key species for stock enhancement based on their biomass potential. Scenario simulations using Ecosim, driven by biomass and fishing factors, indicate that stock enhancement strategies targeting MOB (mussels, oysters, and barnacles) significantly improved the ranching ecosystem, raising its status to “good”. However, the simulations also revealed that stock enhancement had limited effects on optimizing food web structure, system organization, and energy transfer efficiency, suggesting that a combination of strategies is necessary for further improvement.

## Simple Summary

Assessing the structure and function of marine ranching ecosystems is crucial for their successful development. In this study, we used energy flow models and an evaluation system to measure the food web structure and ecological carrying capacity of both a marine ranching ecosystem and a nearby control site. Through this, we identified the most suitable species for stock enhancement and proposed strategies to improve the marine ranching ecosystem. We also used a food web model to simulate the effects of these strategies. Our results show that the marine ranching ecosystem was in better condition than the control site, which may confirm the success of the ranching efforts. Mussels, large crabs, and Scorpaenidae were identified as key groups for enhancement, which could help improve the overall health of the ecosystem. However, we also found that simply increasing the number of a few species is not enough to fully optimize the ecosystem. To improve the food web and the ecosystem as a whole, more comprehensive strategies are needed, such as enhancing protection and management practices, improving artificial reef design and deployment, and boosting ecological connectivity.

## 1. Introduction

Inshore water areas within an Exclusive Economic Zone (EEZ) extend up to 200 nautical miles from the coastal baseline and include both the water column and seabed. These areas are crucial for providing essential ecological services, such as food and raw materials, environmental purification, climate regulation, and cultural enrichment [1,2]. Although they cover only 18% of the Earth’s surface, these areas contribute to 25% of the planet’s primary productivity and account for 90% of global fishery yields [3]. In particular, inshore waters are of vital importance to China, playing a critical role in ensuring food security and maintaining ecological balance [4,5]. Ecological balance is defined as the stable state achieved by ecosystems through development and regulation, characterized by structural stability, functional stability, and a balance in energy input and output [6]. This dynamic equilibrium is crucial as it supports continuous energy flows and material cycles, alongside the renewal of biological entities. However, recent human activities, such as industrialization, urbanization, agricultural expansion, and climate change, have severely degraded these systems. The resulting disruption to China’s inshore habitats and fishery resources has led to reduced fishery yields, diminished biodiversity, and the simplification of food webs [7]. These changes significantly undermine the ecological balance that is essential for sustaining both the productivity and biodiversity of these critical areas.

In response to the challenges posed by the degradation of inshore ecosystems, China has proposed the establishment of a marine ecological civilization and implemented a range of measures aimed at protecting and restoring marine ecosystems. These initiatives include the restoration of nearshore environments, the development of marine ranching projects, the enforcement of seasonal fishing moratoriums, and the regulation of fishing vessels—efforts designed to enhance the stability, diversity, and sustainability of marine ecosystems [8,9]. Among these, the development of marine ranching projects is viewed as a key strategy for transforming and upgrading China’s marine fisheries while safeguarding the marine environment [10].

In China, marine ranching is defined as a fishery model in a specific sea area aimed at rebuilding fishery stocks through measures such as releasing cultured juveniles and restoring habitats while aligning with natural ecosystem processes [11]. It differs from traditional aquaculture and hatchery release. Traditional aquaculture focuses on increasing output through external inputs, emphasizing production and economic benefits, whereas marine ranching emphasizes ecosystem restoration, fully utilizing natural productivity, and prioritizing ecological protection. Compared to pure stock enhancement through hatchery release, marine ranching places greater emphasis on habitat restoration and resource management, which is more conducive to increasing survival and recapture rates of the targeted species [10,11].

The similarities between marine ranching in China and other countries lie in their shared efforts to enhance marine fishery resources and restore ecological environments. The primary methods used include artificial reefs and hatchery release, with a focus on boosting fishery resources and protecting the environment. For example, Japan, South Korea, and the United States enhance marine fishery resources through the deployment of artificial reefs, construction of seaweed beds, and hatchery release; Australia focuses on restoring seagrass beds and coral reefs to conserve marine biodiversity [12]. The differences, however, primarily lie in the industrial models. In Japan and South Korea, marine ranching integrates hatchery release and artificial reef deployment with the establishment of fisheries management centers to enhance fishery resource output. In North America, artificial reef deployment is followed by the development of a large recreational tourism industry, including activities such as diving tourism. European marine ranching, represented by countries like Norway, Germany, and the UK, focuses on hatchery release to increase fishery resources, thereby supporting commercial harvesting [13,14,15,16]. In contrast, China is dedicated to developing a comprehensive industrial framework for marine ranching that integrates site selection, planning, habitat restoration, biomass conservation, and safety assurance, with the aim of enhancing marine fisheries and safeguarding the marine environment. Considerable emphasis has also been placed on the integration of primary, secondary, and tertiary sectors, particularly through the convergence of fisheries and tourism, as well as the alignment of energy and fisheries management [12,17].

Effective assessment of the structural and functional characteristics, as well as the carrying capacity of marine ranching ecosystems, is a fundamental prerequisite for the successful construction and management of marine ranching in China [7]. This is not only crucial for holistic planning but also for ensuring the scientific construction and management of these ecosystems [11,18,19]. Specifically, these assessments provide essential data to guide decisions on the construction area, type, and scale of marine ranching projects. For example, they help in selecting appropriate hatchery release species, estimating optimal stocking densities, and determining the appropriate scale for artificial reef construction [20,21,22,23,24]. Moreover, these assessments are critical for ensuring the long-term sustainability of marine ranching through effective management, which includes determining strategies for sustainable harvest based on carrying capacity [25,26,27].

Because of the limitations in carrying-capacity assessment technology, most marine ranching projects in China have not conducted comprehensive evaluations of carrying capacity [7]. Existing assessments have largely focused on community structure, water quality, and the enhancement effects of individual species [23,28,29,30,31,32,33], with little attention to the overall performance of marine ranching systems [11,34]. Even system-level evaluations generally compare marine ranching with small adjacent areas, lacking a robust evaluation system and clear grading standards [33,35,36,37,38,39], which undermines the credibility of the results. As a result, most marine ranching projects rely heavily on empirical knowledge, lacking a solid scientific foundation for selecting hatchery release species, estimating stocking densities, and designing habitat construction strategies. This reliance leads to significant ecological and economic risks, including low survival rates of stocked species, declines in wild population biomass, depletion of fishery resources, artificial reef subsidence, and hypoxia, as well as wasted financial resources and economic losses due to the high mortality of stocked species [7,10,11,23,40,41,42,43]. Therefore, there is an urgent need for the development of systematic evaluation and simulation methods for marine ranching ecosystems to enhance the scientific rigor and sustainability of marine ranching projects in China.

Ecological Network Analysis (ENA) is a systems-based methodology that quantifies the structure and function of marine ecosystems by analyzing the material and energy flow relationships among all components within ecosystems’ food web [44,45,46,47,48,49,50,51,52]. This approach has proven to be a valuable tool for assessing ecosystem health and for linking ecological and socio-economic systems. It is widely applied in marine ecosystem management and ecological restoration, providing critical insights for decision making and policy development [53,54,55,56].

The Ecopath with Ecosim (EwE) model is widely used to calculate ENA indices for marine ecosystems. It consists of the following three main components: Ecopath, Ecosim, and Ecospace. Ecopath is primarily employed to analyze the material and energy flows among various ecosystem components, while Ecosim simulates the dynamics of ecosystem food webs under different pressures. The EwE model has been extensively applied in recent studies to assess and simulate marine ranching ecosystems [33,35,37,39,57].

In this study, a marine ranching ecosystem and its control counterpart in the Beibu Gulf of China were selected as research subjects. Ecopath models were constructed for both ecosystems to evaluate their energy flow, trophic structure, and ecological carrying capacity. An index system based on ENA and a fuzzy comprehensive evaluation model was developed to assess the status of these ecosystems. Suitable stock enhancement groups for the marine ranching ecosystem were identified, and ecosystem dynamics were simulated under different stock enhancement strategies. This research aims to provide essential technical and theoretical support for the comprehensive evaluation and ecosystem-based stock enhancement of marine ranching. Furthermore, it seeks to contribute to the sustainable and high-quality development of marine ranching in the Beibu Gulf and across China.

## 2. Materials and Methods

### 2.1. Introduction of the Marine Ranching

**Figure 1:**
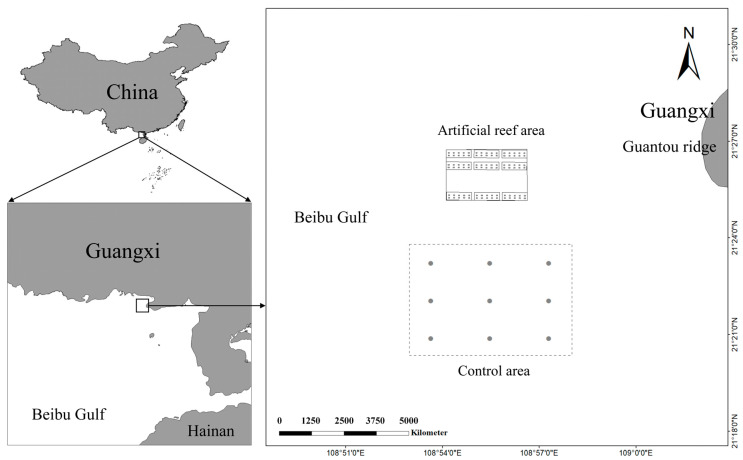
Geographic location and sampling sites of the Jinggong marine ranching and control ecosystems in the Bay of Beibu gulf.

**Figure 2:**
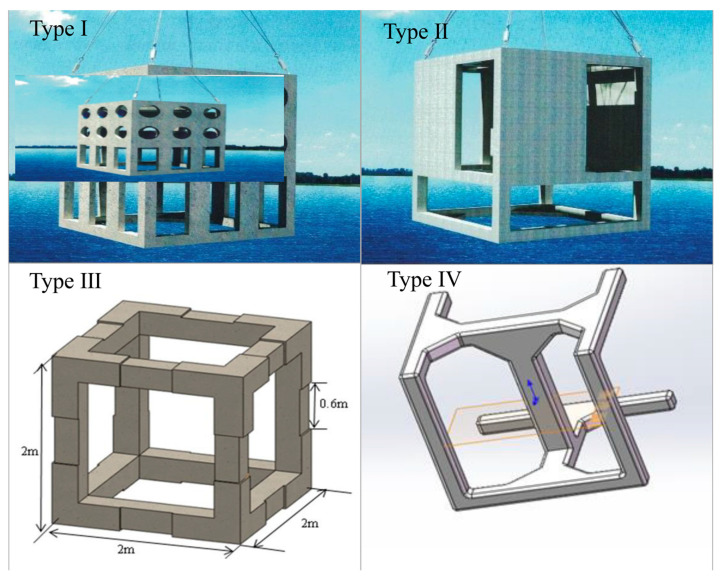
Types of artificial reefs deployed in marine ranching (sizes—type I: 3.0 m × 3.0 m × 3.5 m; type II: 3.0 m × 3.0 m × 3.5 m; type III: 2 m × 2 m × 2 m; type IV: 2.1 m × 2.1 m × 2.1 m).

The marine ranch, named “Jinggong”, is located to the southwest of the Guantou Ridge in Beibu Gulf, with coordinates ranging from 21°25′31.26″ N to 21°25′58.96″ N and from 108°54′15.39″ E to 108°56′38.37″ E. The specific location of the marine ranch is illustrated in Figure 1. Covering an area of 4.80 km^2^, the marine ranch has an average water depth of approximately 9 m. According to the classification standards of the Ministry of Agriculture and Rural Affairs for national marine ranches, the Jinggong Marine Ranch is categorized as a “recreational” type. It was primarily established through artificial reef construction and hatchery releases. Moreover, an online environmental monitoring system was implemented to track the water quality. Since December 2020, approximately 20,000 cubic meters of artificial reefs were deployed, categorized into four types, as shown in Figure 2, with the materials predominantly being composed of reinforced concrete. The primary objective of the marine ranching project is to enhance the conservation and augmentation of fishery resources, thereby ensuring the sustainable development of fisheries. The target species for conservation and enhancement include fish such as Yellowfin Seabream (*Acanthopagrus latus*) and False kelpfish (*Sebastiscus marmoratus*), crustaceans like the Japanese Prawn (*Marsupenaeus japonicus*) and Japanese Stone Crab (*Charybdis japonica*), and Sea cucumber (*Stichopus variegatus*), as well as mollusks such as the Pacific Oyster (*Crassostrea gigas*) and Small Giant Clam (*Lutraria sieboldii*). Additionally, the project aims to stimulate the economy through recreational tourism activities, ultimately promoting the integrated development of both economic and social benefits. The control ecosystem is located about 2 km south of the marine ranch and has a water depth of about 9 m and a sandy silt bottom substrate. The selection of the control site was based on its ecological similarity to the marine ranching, particularly in terms of proximity to land and environmental characteristics. Both areas are located on the western side of Guantou Ridge and exhibit similar water quality conditions. While the ecological features of these areas are comparable, it is recognized that there are still some differences in factors such as flow fields and human activity impacts, particularly due to the ranching area’s closer proximity to the northern mainland.

### 2.2. Construction of Ecopath Models

#### 2.2.1. Introduction of the Ecopath Model

Please refer to the Appendix A: Introduction of Ecopath model.

#### 2.2.2. Survey of the Marine Environment

Surveys of the biotic and abiotic environments in the Jinggong marine ranching and control ecosystems were conducted in spring and autumn of 2023. Phytoplankton biomass was assessed by measuring chlorophyll a according to standard procedures [58]. Zooplankton samples were obtained through vertical tows using plankton nets with a mesh size of 169 µm.

Benthic and swimming organisms in mud substrate areas were surveyed following the Specifications for Marine Surveys GB/T12763.6-2007 [59]. Meanwhile, the biomass of swimming animals (e.g., fish and cephalopods) was calculated using the swept-area method [60]. The hauling speed was approximately 2.5 knots, with an inner mesh size of 3.5 cm and a mouth width of 6 m. Each trawl lasted for 30 min. There were 3 survey sites in the marine ranch and 9 survey sites in the control area.

Swimming organisms and macrobenthos in reef areas were surveyed using SCUBA diving techniques (GoPro, San Mateo, CA, USA) for video recording and hand collection [37,61]. Meanwhile, the survey of fish in the reef area was conducted using a transect method combined with cage net sampling. After the diver descended into the water, they performed transect-based swimming video recording. The video duration at each station was 15 min, with an average sweeping area of 18 square meters per station. Biomass was calculated as the number of each fish species observed multiplied by the average weight derived from the cage net, divided by the swept area of the video recording. Those same transects were used for mobile invertebrates (mainly crustaceans and cephalopods). Macrobenthos such as sea urchins and gastropods were collected from reefs using 0.5 × 0.5 m quadrats, while attached organisms, such as mussels, barnacles, and oysters, were collected by scraping with a small knife. Six stations were set in the reef area. Trawl surveys were also conducted around the reef area; this was primarily aimed at obtaining the biomass of species such as small shrimp, which are difficult to detect and capture with video and cage nets. The taxa, biomass, and abundance of zooplankton, benthic, and swimming animals were measured for all collected samples.

#### 2.2.3. Functional Group Division

Adhering to the functional group division principle of the Ecopath model [62], the marine ranching ecosystem was categorized into 23 functional groups, including pelagic fishes, large and medium demersal fishes, sparids, leiognathidae, small demersal fishes, scorpaenidae, gobiidae, mantis shrimps, large crabs, other crabs, *Metapenaeopsis barbata*, other shrimps, cephalopods, sea urchins, gastropods, barnacles, oysters, mussels, other bivalves, other benthos, zooplankton, phytoplankton, and detritus. The phytoplankton group served as the primary-producer group, while all other groups functioned as consumers, except for detritus. In contrast, the control ecosystem comprised 19 functional groups, which exhibit similar compositions to the marine ranch but excluding sparids, barnacles, oysters, and mussels. The specific species of each functional group are listed in Appendix E: Tables (Table A1 and Table A2).

#### 2.2.4. Data Sources of Functional Groups

The Ecopath models (Ecopath with Ecosim 6.6.7) were designed to model a period of 1 year. The biomass for each functional group is expressed as the wet weight in t/km^2^. The input data for Biomass (B), Production-to-Biomass ratios (P/B), Consumption-to-Biomass ratios (Q/B), and diet composition of each group were estimated using data obtained from field surveys and literature sources. The methods employed to acquire these data are detailed in the Appendix E: Tables (Table A3). The diet composition of each consumer group was displayed in Table A4 and Table A5. The Unassimilated Ratio of Consumption (Ui) for mussels, barnacles, oysters, and other bivalves was set at 0.40, while the group of other benthos was set at 0.35, and all other consumer groups were set at 0.20 [62,63,64]. Fishery data were provided by the Qinzhou Agriculture and Rural Bureau of Guangxi Zhuang Autonomous Region.

#### 2.2.5. Model Balancing and Uncertainty

We used the estimated Ecotrophic Efficiency (EE) value of each functional group (which should be <1) as the primary criterion for model calibration. If the estimated EE exceeded 1, indicating that the consumed biomass surpassed the produced biomass, we incrementally adjusted the diet composition of each consumer group, with each adjustment not exceeding 0.05, to reduce the EE value below 1. Furthermore, we ensured that most of the P/Q values (the gross food conversion efficiency: ratio between production and consumption) were in the range of 0.1–0.3. We also ensured that the Respiration-to-Assimilation (R/A) and Production-to-Respiration (P/R) ratios in the model were <1; the Respiration-to-Biomass (R/B) ratio was higher in active species than in sedentary groups [62,65,66]. The pre-balanced diagnosis was also used to identify issues in the model’s structure and in the data quality prior to balancing the models [65].

### 2.3. Construction of Indices System

#### 2.3.1. Description of ENA Indices

This study established an index system to evaluate the ecosystem status of marine ecosystems based on ENA indices. The ENA indices were categorized into three groups. Firstly, indices representing the function of the ecosystem included Detritivory/Herbivory (D/H), the average Transfer Efficiency among different trophic levels (TE), and relative Ascendancy (A/C). Secondly, indices representing the characteristics of the ecosystem food web’s structure include the Connectance Index (CI), System Omnivory Index (SOI), Finn’s Cycling Index (FCI), and Average Path Length (APL) [67]. Lastly, indices representing the maturity of the ecosystem include Total Primary Production/Total Respiration (TPP/TR), Total Primary Production/Total Biomass (TPP/TB), and Total Biomass/Total System Throughput (TB/TST) (Table 1). The meanings of each index are described in Appendix C: Introduction of Ecological Network analysis indicators.

#### 2.3.2. Classification of Ecosystem Status Levels

Due to the lack of historical data on ENA indices in the study areas, directly evaluating the current status of the ecosystems was challenging. An alternative approach was to establish standards through inter-ecosystem comparison. Therefore, published literature on ecosystem assessments based on ENAs for the world’s coastal marine ecosystems (139 ecosystems in total, including estuaries, bays, islands, straits, oyster reefs, and artificial reefs) was collected, as much as possible, and is presented in Appendix E: Tables (Table A6). The ecosystems were divided into 5 levels using the quintiles method. For positive-type indicators, where a higher value indicates a better system performance, the first, second, third, fourth, and fifth quintiles denote the critical values for the “poor”, “relatively poor”, “medium”, “relatively good”, and “good” grades, respectively. For negative-type indicators, where a higher value indicates a worse system performance, the first, second, third, fourth, and fifth quintiles denote the critical values for the “good”, “relatively good”, “medium”, “relatively poor”, and “poor” grades, respectively (Table 2).

Among the indices, D/H, TE, TB/TST, CI, SOI, FCI, and APL indicate a better ecosystem status as their values increase. Regarding A/C, Ulanowicz et al. (2009) proposed that the optimal trade-off value is 0.4596 [68]; values below this threshold are positively correlated with an improved system performance. Since none of the cases examined in this study exceeded this threshold, A/C was considered a positive indicator. For TPP/TR and TPP/TB, values greater than 1 suggest a lower ecosystem maturity. The weighting of the indicators follows the methodology outlined by Zeng et al. (2021) [69]. As some of the Ecosim scenarios in our study did not include fishing activities, we excluded the mean TL of the catch indicator, as considered by Zeng et al. (2021) [69], and proportionally redistributed its weight among the remaining ecosystem function indices.

#### 2.3.3. Evaluation of the Ecosystem Status of the Ecosystems

Fuzzy Comprehensive Evaluation (FCE) was used to calculate the composite score of all indicators. The final evaluation result was determined based on the principle of maximum membership degree. The specific evaluation steps are referenced from the methods of Tobor-Kapłon et al. (2007), Dong et al. (2021), and Wu and Hu (2020) [70,71,72].

### 2.4. Evaluation of Ecological Carrying Capacity

Ecological carrying capacity is defined as the maximum biomass that functional groups within an ecosystem can sustain while maintaining an energy balance [73]. Specifically, energy balance refers to a state in which the energy inputs and outputs within an ecosystem are in equilibrium [74]. The method for evaluating the ecological carrying capacity was adapted from Jiang and Gibbs (2005) [75]. The biomass of the target functional group was incrementally increased until the ecosystem became unbalanced. The critical point immediately before ecosystem disbalance was identified as the ecological carrying capacity. No parameters other than the biomass of the target functional group were manually altered during the calculation of the ecological carrying capacity.

### 2.5. Evaluation of Stock Enhancement Potential and Selection of Stock Enhancement Groups

All functional groups in the marine ranch were categorized into the following three groups based on their Trophic Levels (TLs): TL 2.0–2.5, TL 2.5–3.0, and TL 3.0–3.5. The difference between the ecological carrying capacity and the current biomass of each functional group was used to assess its stock enhancement potential, with a larger difference indicating higher potential. Within each TL category, groups with higher stock enhancement potential were identified as candidates for stock enhancement. However, the maturity of relevant enhancement technologies, such as seedling breeding and larval releasing, was also considered critical for the final selection. Groups with high enhancement potential but underdeveloped enhancement technologies were excluded from the stock enhancement groups, and only those groups with both high enhancement potential and mature enhancement technologies were selected.

### 2.6. Simulation of Stock Enhancement Strategies

The Ecosim model was employed to simulate the dynamics of the ecosystem food web over the next 14 years under different stock enhancement strategies. In each simulation scenario, the Ecopath model from the tenth year was extracted to represent the new state of the ecosystem. The ecosystem status was then evaluated using the indices system based on the Ecopath model’s ENA indices.

#### 2.6.1. Introduction of the Ecosim Model

Please refer to Appendix B: Introduction of the Ecosim Model.

#### 2.6.2. Construction of Ecosim Model

The Vulnerability (*v*)-index is a critical parameter in constructing the Ecosim model. Which determines whether the trophic control between predator and prey is a top-down, bottom-up, or intermediate effect. Because of the lack of historical survey data in the marine ranch, the following empirical formula was applied to calculate the *v* index for each functional group [76,77]:

(1) $$v_{i}=0.1515\times TL_{i}+0.0485$$

where *TL_i_* is the *TL* corresponding to functional group *i*. The *v* settings ranged from 0 to 1, with 0.0–0.3 representing bottom-up control, 0.3 representing mixed control, and 0.3–1.0 describing a top-down impact [78]. The *v_i_* was then transformed to derive *v_new_* for Ecosim input, which ranged from 1 to ∞, as follows:

(2) $$\log\left(v_{new}\right)=2.301958\times v_{i}+0.001051$$

Within the marine ranching ecosystem, oysters, barnacles, and mussels act as ecosystem engineers, enhancing the spatial complexity and heterogeneity of the habitat. They provide essential refuges, foraging, and reproductive spaces for organisms in the artificial reef area, which in turn influence the behavior and distribution of predators. These effects were incorporated into the simulations using the mediation functions in Ecosim. Sigmoidal functions were used to modulate the predator–prey relationships through these mediation functions in this study. The specific mediation settings were determined based on methodologies outlined by Harvey (2014) and Sadchatheeswaran et al. (2020) [79,80].

#### 2.6.3. Simulation Scenario Design

We assumed that the maximum biomass achievable by the selected stock enhancement group within the marine ranching ecosystem corresponds to the biomass at the ecological carrying capacity. Therefore, setting the biomass of the stock enhancement group at the ecological carrying capacity became one of the driving factors in establishing the Ecosim model. Additionally, fishing effort was also taken into account as a driving factor for the model’s construction. The fishing data used to drive the model were obtained from the fishing efforts in the marine ranch during 2023.

Based on the selection of the stock enhancement groups, the following three species were chosen: mussels, large crabs, and scorpaenids. The method for enhancing mussels in the marine ranch involved constructing artificial reefs, which would provide them with a suitable habitat. However, once the reefs were deployed, not only mussels but also oysters and barnacles grew on them, as these three groups share similar ecological characteristics. As a result, the groups being enhanced were these three. To better simulate the effect of this enhancement method, we included all three of these groups in the enhancement simulation and combined mussels, oysters, and barnacles (MOB) into a single stock-enhancement group.

Three single-group stock enhancement strategies were established for MOB, large crabs, and scorpaenidae. Additionally, the following four multiple-group stock enhancement strategies were set: MOB + large crabs, MOB + scorpaenidae, large crabs + scorpaenidae, and MOB + large crabs + scorpaenidae. For each stock enhancement strategy, the following two scenarios were simulated: with fishing and without fishing.

For with fishing activity, a constant fishing effort was applied throughout the simulation, remaining unchanged over time. The fishing effort was set to match the catches from the marine ranch in 2023. Given that the marine ranching ecosystem had reached a relatively stable state by 2023, this catch level is considered a reasonable reflection of the fishing pressure during the stock enhancement process.

For without fishing activity, the fishing effort was completely excluded in this scenario, and stock enhancement was simulated without any fishing pressure. This allowed for the evaluation of the effects of stock enhancement in the absence of fishing activity.

An additional simulation scenario was also included, involving only fishing activity, where fishing effort was applied without any stock enhancement. Therefore, a total of 15 simulation scenarios were conducted in this study.

## 3. Results

### 3.1. Trophic Structure

The TLs of the functional groups in the marine ranching ecosystem ranged from 1 to 3.46 (Table 3 and Table 4). Sparids exhibited the highest TL, followed by large- and medium-sized demersal fishes and scorpaenidae (3.42 and 3.27, respectively). Additionally, cephalopods, small-sized demersal fishes, and mantis shrimps also displayed relatively high TLs (3.28, 3.22, and 3.22, respectively). Conversely, oysters and mussels had low TLs, both at 2.02. In the control ecosystem, the functional groups exhibited TLs ranging from 1 to 3.63, with sparids being the highest, followed by large- and medium-sized demersal fishes and cephalopods (3.35 and 3.25, respectively). Scorpaenidae and small-sized demersal fishes also had relatively high TLs (3.30 and 3.16, respectively).

The TL I comprised phytoplankton and detritus, TL II mainly consisted of shellfish, barnacles, shrimps, zooplankton, and sea urchins, while TL III mainly consisted of fish, cephalopods, mantis shrimps, and crabs in both ecosystems. Mussels had the highest biomass in TL II (80.40 t/km^2^), followed by barnacles (30 t/km^2^), while sparids had the highest biomass in TL III (0.33 t/km^2^), followed by large crabs and other crabs (both at 0.30 t/km^2^) in the marine ranching ecosystem. The total biomasses distributed among TLs I, II, and III in the marine ranching ecosystem were 60.29, 153.17, and 1.92 t/km^2^, respectively, compared to 65.67, 7.00, and 1.26 t/km^2^, respectively, in the control ecosystem. The phytoplankton showed the highest biomass in the control ecosystem (24.64 t/km^2^).

### 3.2. Energy Flow Structure

The EE values of the functional groups in the two ecosystems are presented in Table 3 and Table 4. In the marine ranching ecosystem, large crabs, mantis shrimps, and cephalopods exhibited high EE values of 0.92, 0.86, and 0.80, respectively. Conversely, sea urchins, barnacles, oysters, and mussels displayed very low EE values due to their high biomass and lack of predators. Shrimps exhibited the highest EE value (0.96) in the control ecosystem, followed by other bivalves, other crabs, *M. barbata*, and other benthic organisms, with EE values of 0.95, 0.92, 0.91, and 0.89, respectively. Leiognathidae, zooplankton, and detritus had very low EE values (0.01, 0.07, and 0.09, respectively).

**Figure 3:**
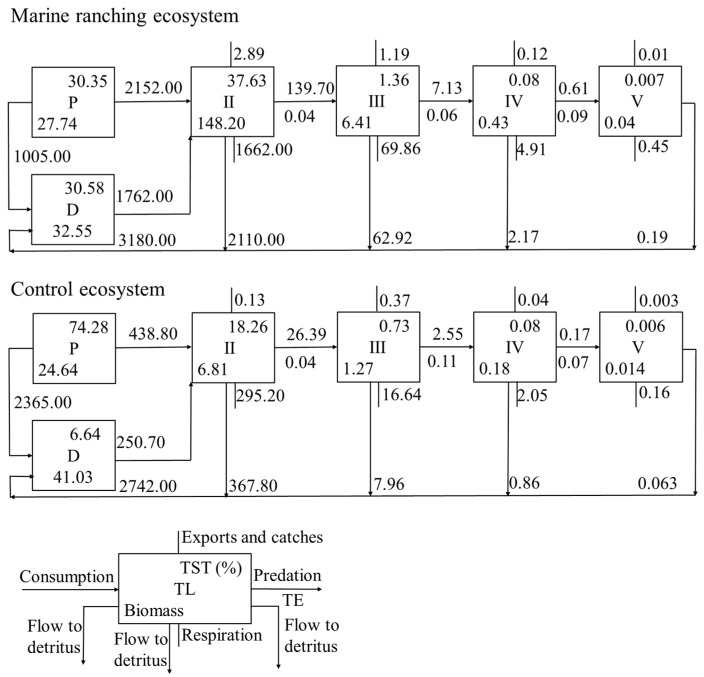
The energy flow (t/km^2^/a) among the different trophic levels in the marine ranching and control ecosystems, respectively (D: detritus; P: primary producers; TL: trophic level; TE: transfer efficiency; TST: total system throughput).

The energy flow among the TLs in the two ecosystems is depicted in Figure 3. Approximately 2152.00 and 2365.00 t/km^2^/a of energy flowed to TL II in the marine ranching and control ecosystems, respectively, accounting for 68.00% and 16.00% of their total primary production, respectively. The transfer efficiencies between TL II and III and III and IV were 4.00% and 6.00% in the marine ranching ecosystem, respectively, they were 4.00% and 11.00%, respectively. The average transfer efficiencies among TLs II to V were 5.84% and 6.47% in the marine ranching and control ecosystems, respectively.

### 3.3. Ecosystem Attributes

The metrics of the Total System Throughput (TST), total production, and total biomass—key indicators of ecological size—were 2.75, 1.40, and 5.56 times higher, respectively, in the marine ranching ecosystem compared to the control ecosystem (Table 5). In the marine ranching ecosystem, the proportion of total consumption to TST was the highest at 39.06%, followed by the flow to detritus, which accounted for 30.56% of the TST. In contrast, in the control ecosystem, the highest proportion was the flow to detritus at 72.59% of the TST, followed by total consumption, which accounted for 19.08%.

### 3.4. Ecosystem Status

The values of the ENA indices of the marine ranching and control ecosystems are presented in Table 6. The evaluated results of the ecosystem statuses of the marine ranching and control ecosystems are presented in Table 7. The ecosystem status of the marine ranching ecosystem was rated as “relatively good”. Specifically, the FCI was classified as “good”, while the FML, TPP/TR, TPP/TB, and TB/TST were classified as “relatively good”. The CI and D/H were classified as “medium”, and the SOI, A/C, and TE were classified as “relatively poor”. In contrast, the ecosystem status of the control ecosystem was rated as “relatively poor”. Specifically, the CI and SOI were classified as “relatively good”, while D/H was classified as “medium”. Furthermore, the FCI, FML, A/C, TPP/TR, and TPP/TB were all classified as “relatively poor”.

### 3.5. Ecological Carrying Capacity and Stock Enhancement Potential

The ecological carrying capacities of the two ecosystems are detailed in Table 8. Notably, the functional groups within the marine ranching system exhibited generally higher carrying capacities compared to those in the control ecosystem. Mussels displayed the highest carrying capacity within the marine ranch at 163 t/km^2^, closely followed by oysters at 96 t/km^2^. Pelagic fishes emerged as the group with the highest carrying capacity among fish species. Conversely, the control ecosystem’s highest carrying capacity was observed with shrimps at 0.95 t/km^2^, followed by large- and medium-sized demersal fishes at 0.48 t/km^2^.

The stock enhancement potential of the economic functional groups within the marine ranching system was estimated based on the disparity between the carrying capacity and current biomass (Table 9). Among groups with TLs of 3.0–3.5, gobiidae exhibited the highest stock enhancement potential, followed by scorpaenidae. In the TL range of 2.5–3.0, pelagic fishes demonstrated the greatest potential, followed by large crabs. For TLs between 2.0 and 2.5, mussels showcased the highest potential, followed by oysters.

Currently, there are no effective stock enhancement technologies available for gobiidae and pelagic fishes. However, seedling breeding and larval releasing technologies for large crabs (mainly composed of *Portunus trituberculatus*) and scorpaenidae (mainly composed of *Epinephelus moara*) have reached a mature stage [81,82], this study opted for large crabs and scorpaenidae as suitable candidates for stock enhancement. Moreover, the enhancement of mussels is primarily hindered by the limited availability of hard substrate within the marine ranch. Therefore, constructing artificial reefs can effectively enhance mussel populations. Recognizing that mussels, oysters, and barnacles (MOB) thrive upon the deployment of artificial reefs, these three groups were amalgamated into a single-stock enhancement group, with an estimated carrying capacity of 218.06 t/km^2^.

### 3.6. Ecosystem Dynamics Under Different Stock Enhancement Strategies

**Figure A1:**
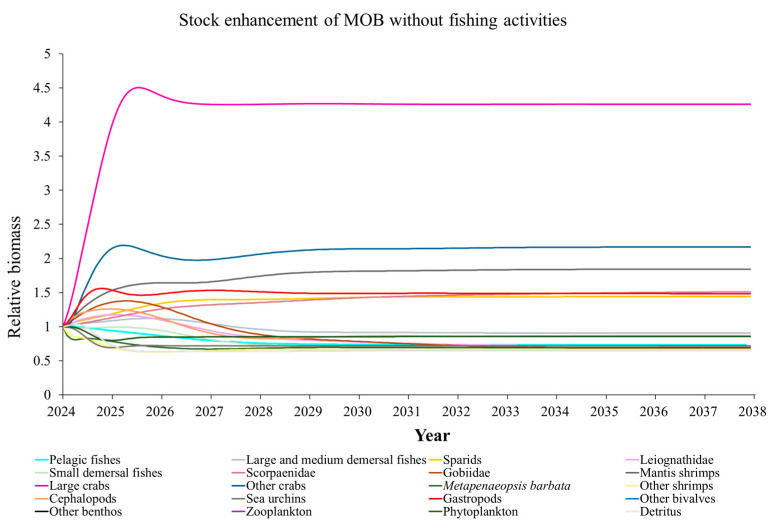
Predicted relative changes in the biomass of each functional group in the Ecopath model of the marine ranching ecosystem for the stock enhancement scenario of MOB without fishing activities. MOB: mussels, oysters, and barnacles.

**Figure A2:**
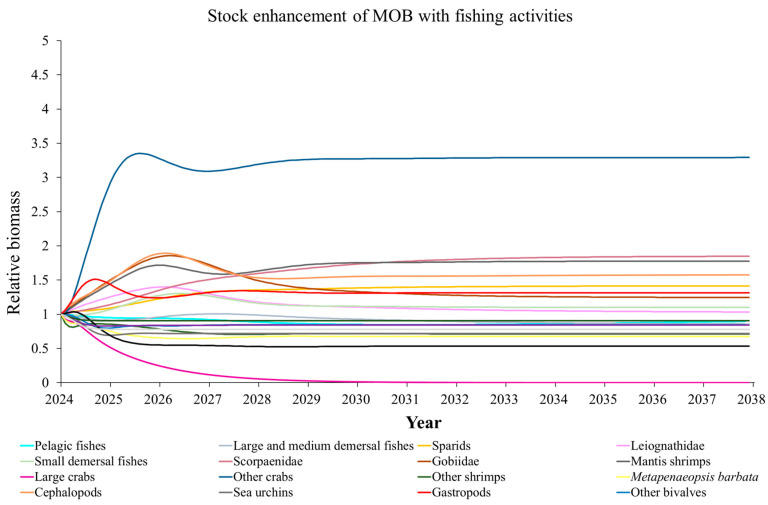
Predicted relative changes in the biomass of each functional group in the Ecopath model of the marine ranching ecosystem for the stock enhancement scenario of MOB with fishing activities. MOB: mussels, oysters, and barnacles.

**Figure A3:**
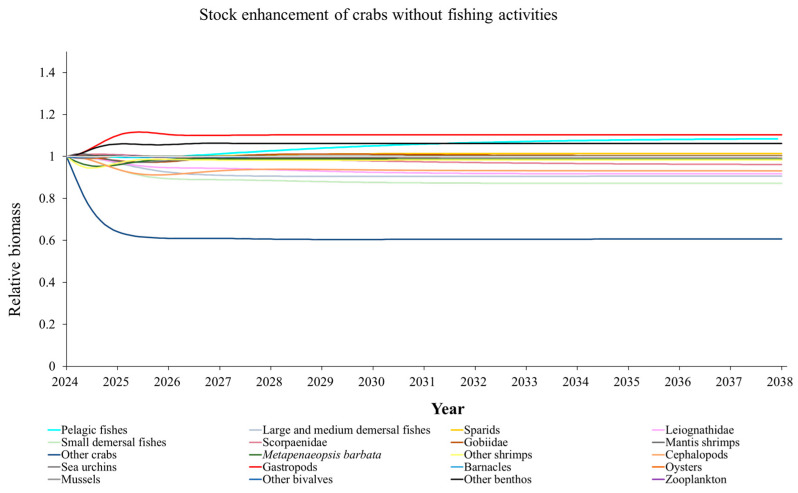
Predicted relative changes in the biomass of each functional group in the Ecopath model of the marine ranching ecosystem for the stock enhancement scenario of crabs (functional group of large crabs) without fishing activities.

**Figure A4:**
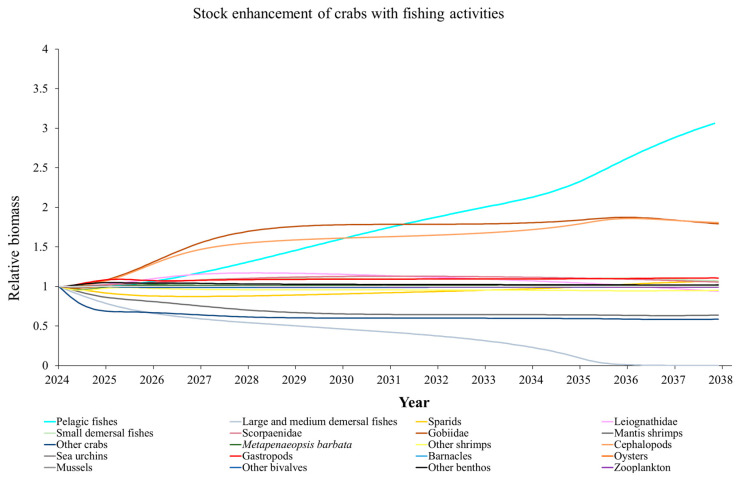
Predicted relative changes in the biomass of each functional group in the Ecopath model of the marine ranching ecosystem for the stock enhancement scenario of crabs (functional group of large crabs) with fishing activities.

**Figure A5:**
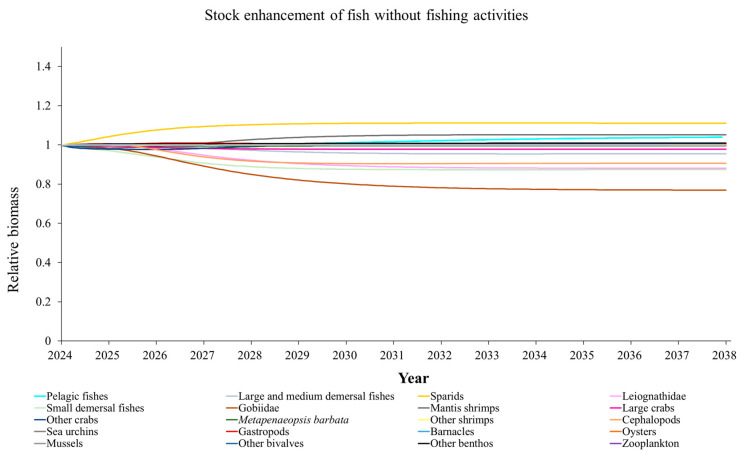
Predicted relative changes in the biomass of each functional group in the Ecopath model of the marine ranching ecosystem for the stock enhancement scenario of fish (functional group of scorpaenidae) without fishing activities.

**Figure A6:**
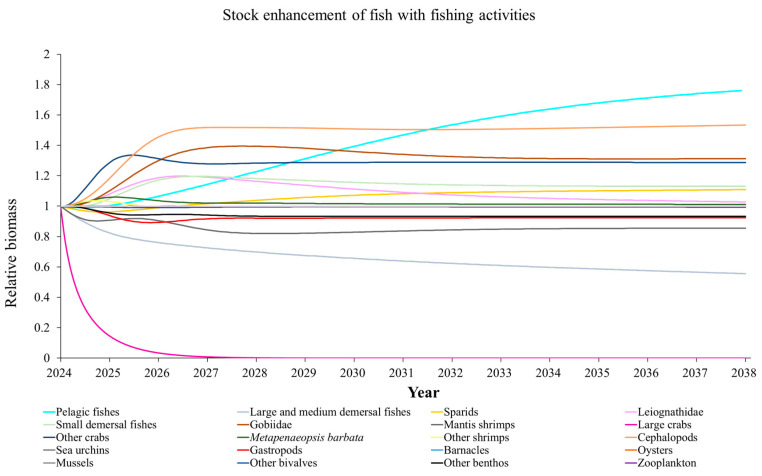
Predicted relative changes in the biomass of each functional group in the Ecopath model of the marine ranching ecosystem for the stock enhancement scenario of fish (functional group of scorpaenidae) with fishing activities.

**Figure A7:**
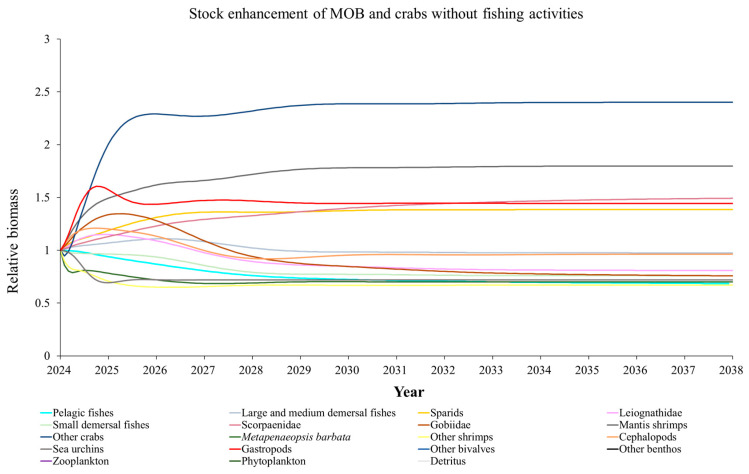
Predicted relative changes in the biomass of each functional group in the Ecopath model of marine ranching ecosystem for the stock enhancement scenario of MOB and crabs without fishing activities.

**Figure A8:**
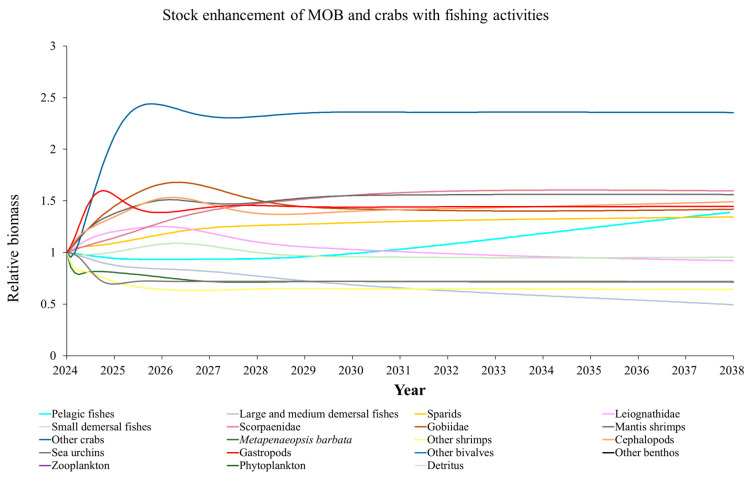
Predicted relative changes in the biomass of each functional group in the Ecopath model of the marine ranching ecosystem for the stock enhancement scenario of MOB and crabs with fishing activities.

**Figure A9:**
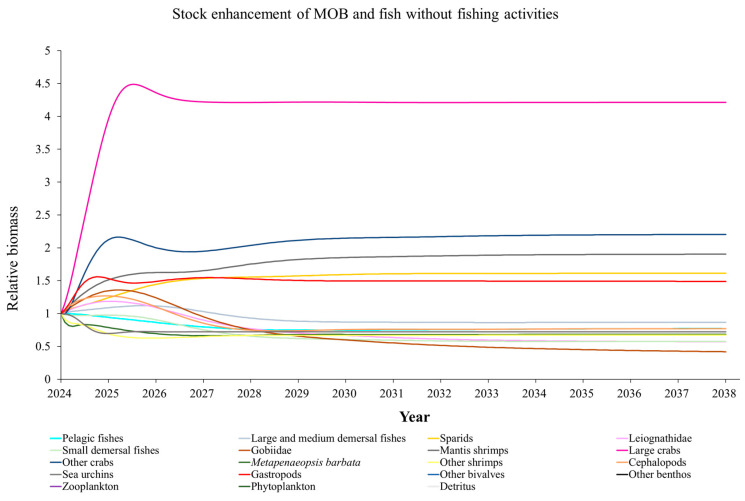
Predicted relative changes in the biomass of each functional group in the Ecopath model of the marine ranching ecosystem for the stock enhancement scenario of MOB and fish without fishing activities.

**Figure A10:**
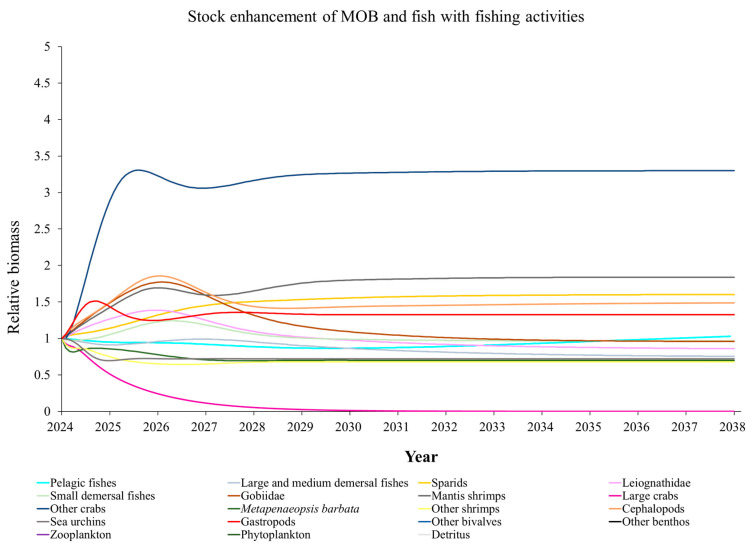
Predicted relative changes in the biomass of each functional group in the Ecopath model of the marine ranching ecosystem for the stock enhancement scenario of MOB and fish with fishing activities.

**Figure A11:**
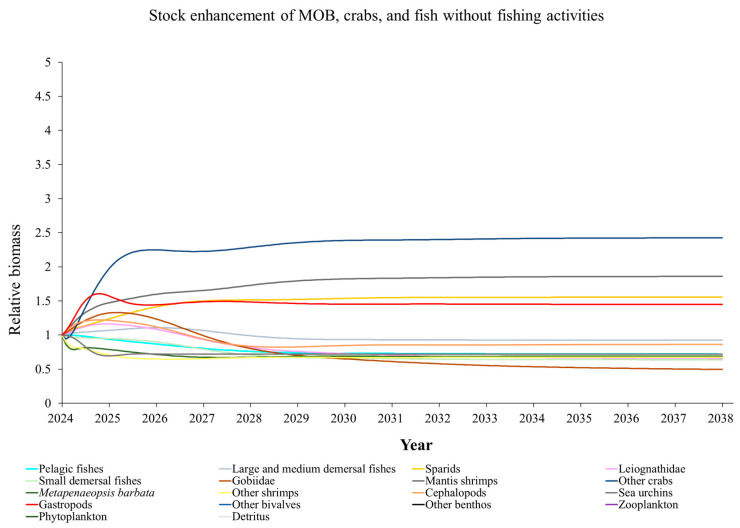
Predicted relative changes in the biomass of each functional group in the Ecopath model of the marine ranching ecosystem for the stock enhancement scenario of MOB, crabs, and fish without fishing activities.

**Figure A12:**
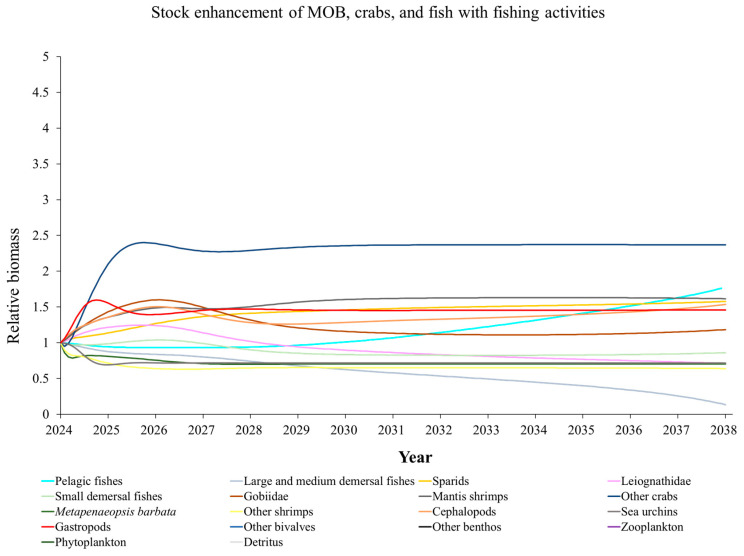
Predicted relative changes in the biomass of each functional group in the Ecopath model of the marine ranching ecosystem for the stock enhancement scenario of MOB, crabs, and fish with fishing activities.

**Figure A13:**
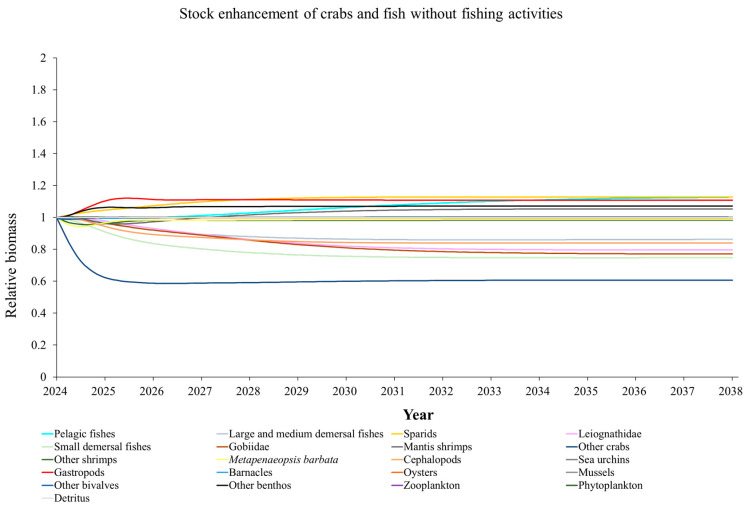
Predicted relative changes in the biomass of each functional group in the Ecopath model of the marine ranching ecosystem for the stock enhancement scenario of crabs and fish without fishing activities.

**Figure A14:**
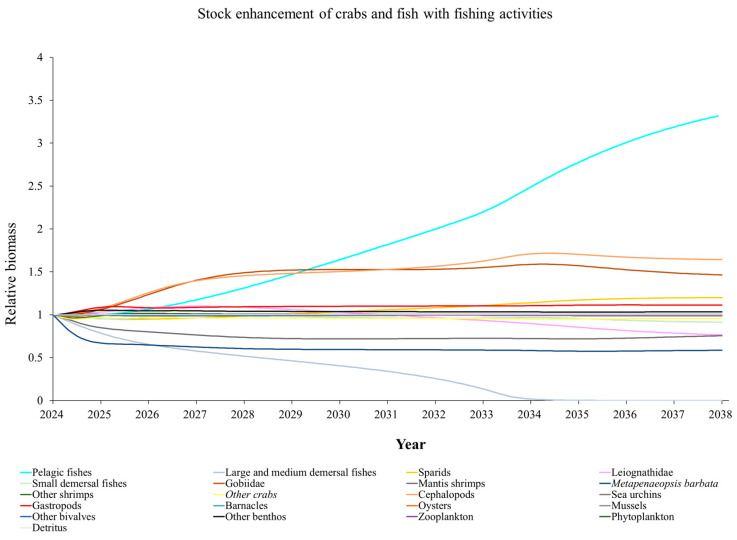
Predicted relative changes in the biomass of each functional group in the Ecopath model of the marine ranching ecosystem for the stock enhancement scenario of crabs and fish with fishing activities.

**Figure A15:**
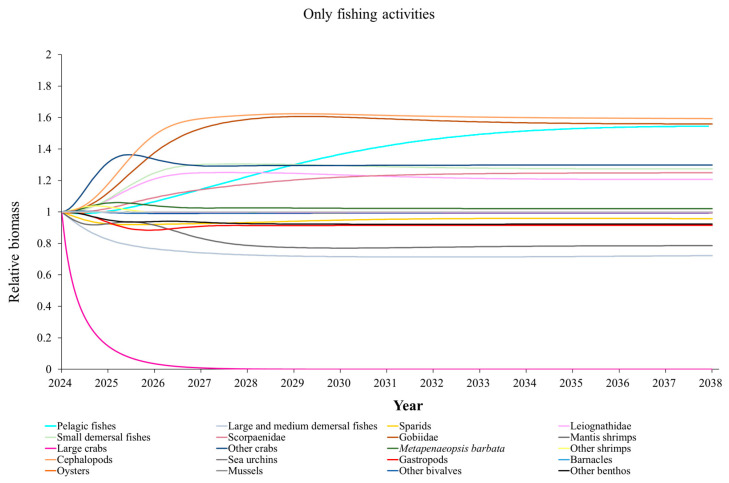
Predicted relative changes in the biomass of each functional group in the Ecopath model of the marine ranching ecosystem for the scenario with fishing activities.

The Ecosim model was employed to simulate the dynamics of the marine ranching ecosystem under various stock enhancement scenarios for the forthcoming 14 years. The outcomes are illustrated in Appendix F: Figures (Figure A1, Figure A2, Figure A3, Figure A4, Figure A5, Figure A6, Figure A7, Figure A8, Figure A9, Figure A10, Figure A11, Figure A12, Figure A13, Figure A14 and Figure A15). Table 10 and Table 11 present the ecosystem status of the marine ranching system at the tenth year of modeling. The results reveal that in scenarios involving fishing alone, stock enhancement of MOB + fishing, scorpaenidae + fishing, and MOB + scorpaenidae + fishing, large crabs experienced a rapid decline in biomass to zero. Similarly, in scenarios of large crabs + fishing and large crabs + scorpaenidae + fishing, the biomass of large and medium demersal fishes also decreased to zero. These observations suggest a collapse in the energy flow structure within these scenarios. Consequently, only the ecosystem statuses of predicted scenarios without such collapse can be effectively evaluated using the index system. The evaluated results indicate that in scenarios involving the stock enhancement of MOB, MOB + large crabs, MOB + large crabs + fishing, MOB + scorpaenidae, MOB + large crabs + scorpaenidae, and MOB + large crabs + scorpaenidae + fishing, the ecosystem status notably improved to a “good” level. Specifically, there was an observed decrease in the indices of TPP/TR and TPP/TB, accompanied by observed increases in the FCI and FML, while other indices displayed minimal changes.

## 4. Discussion

In China, the primary objective behind developing marine ranching was to restore the marine environment and ensure a sustainable yield of fishery resources [83]. Following almost four years of development, the Jinggong marine ranch has experienced significant transformations in both biotic community composition and system functionality compared to the control ecosystem. The biomasses of fish, crustaceans, and mollusks within the marine ranch are 1.82, 16.99, and 33.51 times greater, respectively, than those observed in the control ecosystem.

Artificial reefs play a crucial role in significantly increasing the mollusk biomass by providing essential substrates for attached organisms such as mussels and oysters [84,85,86]. These mussels and oysters, through their robust filter-feeding and biodeposition activities, transfer substantial amounts of particulate organic matter to benthic environments, thereby enriching these habitats with essential nutrients [87]. Furthermore, artificial reefs modify the flow field, creating slowed currents within the reef structure [88,89]. This alteration increases the deposition rate of particulate organic matter and further enhances the nutrient content in the reef’s benthic environment. Consequently, the elevated nutrients stimulate the activity of benthic microorganisms, accelerating the transformation and cycling of nutrients at the water’s bottom [90]. These processes provide additional nutrients for benthic microalgae and enhances their primary productivity [91,92]. Additionally, artificial reefs can generate upwelling, which transports nutrients from the bottom to upper water layers, thus accelerating nutrient cycling and enhancing the primary productivity of pelagic phytoplankton [93,94]. Moreover, the significant spatial heterogeneity provided by artificial shellfish reefs offers effective refuges and foraging grounds for marine organisms [93,95,96]. These functions of artificial reefs—particularly accelerating nutrient cycling, enhancing primary productivity, and providing habitats for protection, foraging, and breeding—are crucial in supporting the growth and reproductive success of various marine organisms. Ultimately, these activities significantly enhance the biodiversity and biomass of marine resources [97,98].

The SOI and CI serve as crucial indicators of ecosystem food web complexity. In the marine ranching and control ecosystems, the CI values were 0.28 and 0.32, respectively, while the SOI values were 0.13 and 0.21, respectively. Comparatively, the CI in the marine ranching system was rated at a “medium” level in relation to those of the coastal marine ecosystems collected in this study, while the SOI was rated as “relatively poor”. Moreover, both the CI and SOI values were notably lower compared to the small marine ecosystems (1–10 km^2^) investigated by Heymans et al. (2014) [51]. The CI reflects the ratio of actual links to potential links within an ecosystem’s food web, whereas the SOI delineates the distribution of feeding interactions across TLs [49,62]. The SOI compensates for the limitations of the CI in delineating food web complexity, especially given that the CI remains constant despite variations in the prey proportions within predator diets [62,99]. The low SOI observed in the marine ranching ecosystem suggests weak connections among functional groups, potentially resulting in lower energy flux and a simplified food web structure. The lower CI and SOI in the marine ranch compared to the control ecosystem may stem from increased biomasses of mollusk, echinoderm, and barnacles. The simpler diets of these species contribute to a less complex food web structure. The increased biomass of these groups also contributed to increased EE values for phytoplankton and detritus within the marine ranching ecosystem, resulting in a notable enhancement of energy utilization efficiency between TLs I and II. However, despite these improvements, the TE values among TLs II to V remained notably low, averaging 5.84%. This level was classified as “relatively poor” according to the index system, falling far below the natural ecosystem average of 10% [100]. Optimizing the food web to facilitate smoother energy transfer pathways and increasing the biomass of the higher TL organisms (such as those at TLs III and IV) are two essential aspects of improving TE values across TLs II to V. Mid-TL organisms, including crustaceans, cephalopods, and bivalves, play a pivotal role as intermediaries in the energy transfer between primary producers and apex predators [101,102,103]. Employing strategies such as habitat restoration, biological conservation, and stock enhancement to increase the diversity and biomass of mid-TL organisms may help optimize the food web and increase the biomass of higher TL organisms, thereby improving the TE values across TLs II to V.

The A/C serves as an indicator of ecosystem organization and efficiency. Both ecosystems in this study were classified at the “relatively poor” level, suggesting diminished organization and system efficiency. The observed low A/C value in the marine ranching ecosystem may be attributed to the fact that increasing system organization levels hinge on the succession of community structure, a process that typically unfolds over an extended period [104,105]. Additionally, external disturbances, such as fishing activities and marine natural disasters, can disrupt the self-organization process of the ecosystem [106]. For instance, massive waves and strong winds caused by typhoons can disturb seabed sediments, leading to a significant decrease in the abundance and species composition of large benthic organisms within a short period [107]. This, in turn, hinders the ecosystem’s ability to achieve higher levels of organization.

The low complexity of the food web, TE, and A/C values in marine ranching suggest that this system may be subject to significant external pressures [51,108,109,110], resulting in the majority of the TST occurring at low TLs. Overfishing emerges as a prominent source of pressure among various external stressors. Indeed, overfishing has led to a severe decline and, in some cases, the depletion of large carnivorous fish in China’s coastal ecosystems [7,111], echoing the phenomenon of “fishing down the marine food web” observed in diverse contexts [112,113,114]. Modeled results from the Ecosim in this study indicate that large crabs in the marine ranch are subject to overfishing. Chen et al. (2008) similarly observed overfishing in the Beibu Gulf [115]. Fishing activities in areas adjacent to marine ranching may adversely affect marine ranching. The migration patterns of many marine creatures within and around these ranches, particularly for types II and III fish species that only spend part of their lifecycle in the ranches, expose them to significant fishing risks during their movement among regions [11,116,117,118]. Considering the diverse habitat requirements of marine creatures at different life stages, proposals such as those by Yang and Ding (2022) are noteworthy [119]. They suggest constructing global aquatic ecological ranching systems that designate entire estuaries or bays as ranching areas. Similarly, Liang et al. (2020) propose developing marine ranching facilities designed to meet all habitat needs across different life stages of fish [118]. In the long term, these proposals hold the potential to mitigate challenges faced by marine ranching, including strong external disturbances and difficulties in maintaining a complex food web structure and high system organization levels.

The TPP/TR and TPP/TB ratios in the marine ranching and control ecosystems were 1.82 and 8.93, and 17.27 and 85.22, respectively. These values in the marine ranch were rated as “good”, whereas those in the control ecosystem were deemed “relatively poor”. Additionally, the D/H ratio in the marine ranch exceeded that in the control ecosystem. These findings suggest that the establishment of marine ranching has significantly enhanced ecosystem maturity and stability [120,121]. Despite comparable total primary production in both ecosystems, the decline in the TPP/TR and TPP/TB ratios can be attributed to increases in the total biomass and total respiration. Notably, in marine ranching, filter-feeding bivalves accounted for the largest portion of both the total biomass and total respiration. These bivalves exhibit ecological characteristics similar to those of zooplankton but with lower turnover rates. While zooplankton can quickly respond to ecosystem disturbances, bivalves exhibit slower responses, thus creating a pathway for slow energy flow. The asynchronous dynamics of bivalves and zooplankton likely play a pivotal role in sustaining ecosystem stability [122].

The evaluation based on the index system revealed that the ecosystem status in marine ranching was “relatively good”, contrasting with the “relatively poor” status of the control ecosystem. Notably, marine ranching’s maturity indices, such as TPP/TR, TPP/TB, and TB/TST, significantly outperformed those of the control ecosystem. Furthermore, indices like the FCI and FML demonstrated marked improvements, indicating that marine ranching enhances system maturity, energy recycling efficiency, and food chain length. Moreover, metrics such as TST, total production, and total biomass in marine ranching were 2.75, 1.40, and 5.56 times higher, respectively, than those in the control ecosystems. The carrying capacities for fish, crustaceans, and shellfish have also significantly increased in marine ranching areas, these may indicate the positive impacts of marine ranching construction efforts. However, despite these achievements, the construction efforts have yet to effectively improve metrics such as the TE, level of system organization, and food web complexity. Although the results indicate positive effects of marine ranching, it is important to interpret these findings with caution. Differences in factors such as terrestrial inputs and flow fields between the ranching and control areas could influence the outcomes. Future studies incorporating multiple control sites would be valuable for a more robust comparison.

Stock enhancement serves as a pivotal approach to improving marine environments and achieving sustainable utilization of fishery resources [123]. However, the efficacy of stock enhancement in restoring marine resources varies across different initiatives [23,124,125,126,127]. Some studies even contend that this method is entirely ineffective [128]. Simulation scenarios conducted in this study revealed that stocking single fish or crab species did not notably optimize the ecosystem. However, the stock enhancement of the bivalve-dominated MOB group enhanced the ecosystem status from “relatively good” to “good”. This may be attributed to the higher enhancing capacity of MOB compared to crab and fish groups. Additionally, the augmentation in biomasses of mussels, barnacles, and oysters expanded shellfish reefs, providing increased refuge and food resources for crustaceans, cephalopods, fish, and other organisms [86]. This indirect effect also contributed positively to optimizing the ecosystem status.

Yang et al. (2023) demonstrated that stock enhancement of multiple species may have better ecological effects than single-species stock enhancement [129]. This finding aligns with the results of our study. For instance, in scenarios involving single-group stock enhancement of crab, fish, and the MOB group alongside fishing activities, the populations of large crabs or large and medium demersal fishes rapidly collapsed. Conversely, in scenarios combining MOB with crab and fishing, or MOB with crab, fish, and fishing, all functional groups maintained relatively stable biomasses, leading to an increase in ecosystem status to a higher level (from “relatively good” to “good”). These findings suggest that stock enhancement involving multiple species from different TLs represents a more effective strategy for optimizing ecosystem structure and function.

It is important to note that maintaining the biomass of stock enhancement groups at the group’s carrying capacity in simulation scenarios may not fully reflect real-world conditions. The first point is that the estimated ecological carrying capacity was derived based on specific assumptions. The carrying capacity was calculated using a static energy flow model, which represents a theoretical value derived from an ecosystem’s energy balance perspective. This model does not account for the dynamics of biological growth and migration within different functional groups, nor does it consider the impacts of environmental fluctuations [38], Particularly in ecosystems where the biomass or density of a single species increases significantly, issues such as disease outbreaks and interspecies competition may arise [130,131,132,133]. Additionally, the marine ranching area is located near the coast, with a water depth of approximately nine meters. This may support the relatively high primary productivity of benthic microalgae [134]. However, the Ecopath model established did not consider the primary productivity of benthic microalgae, leading to an underestimation of the overall ecosystem’s primary productivity, as well as the carrying capacity of the groups that feed on benthic microalgae. Moreover, the carrying capacity of each functional group is dynamically influenced by external factors, such as climate change and human activities [25]; therefore, it is essential to consider the dynamic nature of carrying capacity in real-world stocking activities [24,38,135]. These factors may lead to discrepancies between the estimated carrying capacity and the actual situation.

The second point is that the instability of the ecosystem increases as it nears its carrying capacity, which makes its dynamic changes difficult to predict. As ecosystems approach this limit, resource constraints, such as prey availability and habitat space, become more pronounced, potentially diminishing their resilience. These increased limitations may heighten the ecosystem’s sensitivity to disturbances, leading to greater population volatility and instability [136,137,138]. Under such conditions, even minor external disturbances or internal fluctuations could push the ecosystem into a state of energy imbalance, significantly elevating ecological risks.

Consequently, these might lead managers to engage excessively in stock enhancement activities, thereby propelling the ecosystem toward a state of energy disequilibrium. This disequilibrium could, in turn, precipitate significant mortality events within the enhanced species and result in the collapse of other functional groups. Therefore, when conducting stock enhancement activities, adopting cautious adaptive management strategies is essential [17]. Under such strategies, integrating the ecological roles of various organisms, implementing multispecies stock enhancements [127], or reducing the stocking density of target organisms may help mitigate these risks.

This study aims to conduct stock enhancement with the objective of maintaining the biomasses of the enhanced groups at their carrying capacity. However, while this strategy maximizes the biomasses of the target groups, factors such as resource availability (e.g., food and space) and disease may prevent the attainment of the Maximum Sustainable Yield (MSY). According to Schaefer (1954), Legović and Perić (1984), and Clark (1990) [139,140,141], MSY is typically achieved when a population reaches half of its carrying capacity. However, this conclusion is based on the assumption of an isolated population growing according to logistic growth models, which overlooks interspecies interactions and food web complexities [142]. As a result, the carrying capacity derived from this approach may exceed the ecological carrying capacity estimated in this study while potentially falling short of the production carrying capacity, which represents the maximum level of production [143,144].

To achieve the MSY for key groups in marine ranching, it may be beneficial to implement appropriate harvesting activities for groups such as sparids and large and medium demersal fishes, whose biomasses are approaching their carrying capacity, thus eliminating the need for stock enhancement. In contrast, species such as large crabs, scorpaenidae, and oysters still maintain a biomass well below half of their carrying capacity, indicating considerable potential for stock enhancement. Therefore, to mitigate the risks associated with such enhancement efforts, it would be prudent to manage the biomass of these groups at approximately half of their ecological carrying capacity.

In scenarios focused on MOB (excluding MOB + fishing and MOB + fish + fishing), the ecosystem status was elevated to a “good” level, indicating that the ecosystem structure and function were effectively optimized compared to the initial state. However, this does not imply that the ecosystem has reached an ideal or perfect state. The index system we constructed in this study provides a general overview of coastal ecosystems, and achieving a ’good’ level does not necessarily align with the standards of mature ecosystems as described by Odum (1969) [120]. For example, the values of TE, A/C, CI, and SOI showed only slight changes in these scenarios, remaining at “relatively poor” or “medium” levels. This indicates that the simulated stock enhancement strategies had a limited impact on optimizing the food web structure, system organization level, and energy transfer efficiency. This finding aligns with the conclusions of Yang et al. (2023) [129], who reported that stock enhancement did not significantly increase ecosystem species diversity, and its restoration effects were limited. It can be inferred that stock enhancement of only a few species may not be sufficient to fully optimize system structure and function. In line with this, Hemraj et al. (2024) emphasized the importance of prioritizing protection and natural recovery to enhance ecosystem structure and function [145]. To truly optimize the structure and function of marine ranching ecosystems, it is crucial to adopt a more integrated approach that includes strengthening ecological connectivity between marine ranching and adjacent areas, improving fishing management strategies, and advancing the design and deployment of artificial reefs [27,146,147,148]. When implemented together, these measures could provide a more sustainable and holistic framework for ecosystem restoration and management in marine ranching.

## 5. Conclusions

In this study, we developed two Ecopath models to assess the energy flow and trophic structure of both marine ranching and control ecosystems. Additionally, we established an index system based on ENA and the fuzzy comprehensive model, incorporating ENA data from 139 coastal marine ecosystems to evaluate the ecosystem status. The results provide valuable insights for the ongoing development of marine ranching initiatives. Importantly, this index system addresses the challenge of lacking reliable standards for evaluating marine ranching ecosystems, offering a methodological framework that can support the assessment of other marine ranching projects in China. The ecosystem status was rated as “relatively good” in the marine ranching ecosystem and “relatively poor” in the control ecosystem. The TST, total production, total biomass, and ecological carrying capacity of economic groups were significantly higher in the marine ranching ecosystem compared to the control, which may indicate the success of the construction efforts. Based on their biomass enhancement potential, mussels, large crabs, and scorpaenidae were identified as the most suitable groups for stock enhancement. Future research should focus on determining the optimal stocking densities by accounting for the mortality rates and growth characteristics of stock enhancement species and exploring the optimal stock levels that would ensure the maximum sustainable yield. The scenario simulations conducted using Ecosim revealed that stock enhancement strategies involving MOB (excluding MOB + fishing and MOB + fish + fishing) elevated the status of marine ranching to a “good” level. Furthermore, enhancing multiple species simultaneously appears to yield better ecological outcomes than focusing solely on single-species stock enhancement. In the simulation scenarios, the biomasses of stock enhancement groups were assumed to reach their carrying capacity levels, which may not fully reflect real-world conditions. Future research should focus on exploring the dynamics of the ecological carrying capacity across different temporal scales and adjusting stock enhancement strategies accordingly to achieve the most effective ecological outcomes. The scenario simulations suggest that stock enhancement involving only a few species is insufficient to fully optimize ecosystem structure and function. To address this limitation, future research should adopt a more integrated approach, focusing on strengthening protection and management strategies, as well as advancing the design and deployment of artificial reefs. These measures could play a pivotal role in enhancing ecosystem structure and function, ultimately ensuring the long-term sustainability and resilience of marine ranching ecosystems.

## Figures and Tables

**Table 1:** Selected indices and their weight assignment.

Overall Target	Criterion Layer	Indicator Layer	Weight
Ecosystem state	Ecosystem function	D/H	0.133
	0.4	A/C	0.133
		TE	0.133
	Food web structure	CI	0.114
	0.4	SOI	0.114
		FCI	0.114
		FML	0.057
	Ecosystem maturity	TPP/TR	0.1
	0.2	TPP/TB	0.05
		TB/TST	0.05

D/H: detritivory: herbivory ratio; TE: transfer efficiency; A/C: relative ascendancy; FCI: Finn’s cycling index; CI: connectance index; SOI: system omnivory index; FML: Finn’s mean path length; TPP/TR: total primary production/total respiration; TPP/TB: total primary production/total biomass; TB/TST: total biomass/total throughput.

**Table 2:** The values of interval points across the different evaluation grades for indicators.

Indicator	Grades					Indicator Type
	Poor	Relatively Poor	Medium	Relatively Good	Good	
D/H	0.073	0.414	0.540	0.631	1.209	Positive
TE	2.920	6.800	9.400	11.500	13.236	Positive
A/C	0.150	0.264	0.302	0.339	0.368	Positive
CI	0.100	0.204	0.265	0.310	0.348	Positive
SOI	0.009	0.144	0.180	0.210	0.271	Positive
FCI	0.650	2.800	4.980	9.400	14.700	Positive
FML	1.206	2.336	2.568	3.193	4.000	Positive
TPP/TR	15.509	3.522	2.572	1.922	1.346	Negative
TPP/TB	132.000	48.656	31.964	17.338	9.110	Negative
TB/TST	0.003	0.008	0.011	0.019	0.030	Negative

The abbreviations are the same as in Table 1.

**Table 3:** The TL, B, P/B, Q/B, EE, and Ui of each functional group in the marine ranching ecosystem.

Functional Group	TL	B (t/km^2^)	P/B (/a)	Q/B (/a)	EE	Ui
Pelagic fishes	2.70	0.11	0.60	16.29	0.76	0.20
Large and medium demersal fishes	3.42	0.18	1.16	12.82	0.77	0.20
Sparids	3.46	0.33	0.95	14.43	0.19	0.20
Leiognathidae	2.86	0.24	2.40	14.12	0.50	0.20
Small demersal fishes	3.22	0.18	1.76	13.60	0.72	0.20
Scorpaenidae	3.27	0.02	0.97	4.00	0.25	0.20
Gobiidae	3.22	0.07	2.32	12.96	0.86	0.20
Mantis shrimps	3.08	0.12	5.98	30.81	0.18	0.20
Large crabs	2.89	0.30	5.49	26.79	0.92	0.20
Other crabs	3.13	0.30	6.82	30.35	0.75	0.20
*Metapenaeopsis barbata*	2.33	0.52	7.89	27.61	0.31	0.20
Other shrimps	2.37	1.50	6.68	23.92	0.63	0.20
Cephalopods	3.28	0.07	3.81	14.83	0.80	0.20
Sea urchins	2.10	11.43	6.38	23.60	0.01	0.20
Gastropods	2.46	0.88	5.75	23.83	0.63	0.20
Barnacles	2.02	30.00	6.15	27.19	0.01	0.20
Oysters	2.02	21.76	4.61	20.93	0.07	0.40
Mussels	2.02	80.40	5.06	19.34	0.05	0.40
Other bivalves	2.02	1.58	6.20	23.46	0.74	0.40
Other benthos	2.34	0.93	6.10	21.95	0.61	0.35
Zooplankton	2.00	4.17	32.54	192.47	0.73	0.40
Phytoplankton	1.00	27.74	113.82		0.68	
Detritus	1.00	32.55			0.55	

TL: trophic level; B: biomass; P/B: production/biomass; Q/B: consumption/biomass; EE: ecotrophic efficiency; Ui: unassimilated consumption.

**Table 4:** The TL, B, P/B, Q/B, EE, and Ui of each functional group in the control ecosystem.

Functional Group	TL	B (t/km^2^)	P/B (/a)	Q/B (/a)	EE	Ui
Pelagic fishes	2.70	0.06	0.60	16.29	0.73	0.20
Large and medium demersal fishes	3.35	0.18	1.30	15.18	0.78	0.20
Sparids	3.63	0.01	0.73	15.87	0.69	0.20
Leiognathidae	2.84	0.18	2.75	17.81	0.01	0.20
Small demersal fishes	3.16	0.12	1.83	12.75	0.61	0.20
Scorpaenidae	3.30	0.00	0.97	4.00	0.16	0.20
Gobiidae	3.16	0.07	2.30	13.92	0.41	0.20
Mantis shrimps	3.01	0.13	5.18	31.68	0.13	0.20
Large crabs	2.84	0.33	4.67	24.38	0.34	0.20
Other crabs	3.09	0.11	4.84	24.90	0.92	0.20
*Metapenaeopsis barbata*	2.31	0.06	7.40	28.04	0.91	0.20
Other shrimps	2.37	0.58	6.77	25.85	0.96	0.20
Cephalopods	3.25	0.07	4.58	15.82	0.36	0.20
Gastropods	2.42	0.26	5.70	23.80	0.65	0.20
Bivalves	2.01	2.86	5.65	23.77	0.95	0.40
Other benthos	2.08	0.11	6.10	21.95	0.89	0.35
Zooplankton	2.00	3.13	32.39	192.29	0.07	0.40
Phytoplankton	1.00	24.64	113.82		0.16	
Detritus	1.00	41.03			0.09	

The abbreviations are the same as in Table 3.

**Table 5:** Ecosystem attributes of the marine ranching and control ecosystems.

Attribute	Marine Ranching Ecosystem	Control Ecosystem	Unit
Total system throughput	10,404.99	3777.48	t/km^2/^a
Total consumption	4064.63	720.83	t/km^2^/a
Total export	1422.78	0.54	t/km^2^/a
Total respiration	1737.31	314.03	t/km^2^/a
Total flow to detritus	3180.26	2742.08	t/km^2^/a
Total production	4098.69	2932.48	t/km^2^/a
Total primary production	3157.37	2804.18	t/km^2^/a
Total biomass	182.83	32.90	t/km^2^

**Table 6:** The values of the ENA indicators in the marine ranching and control ecosystems.

Ecosystem	ENA Indicators									
	Ecosystem Function			Food Web Structure				Ecosystem Maturity		
	D/H	TE	A/C	CI	SOI	FCI	FML	TPP/TR	TPP/TB	TB/TST
Marine ranching	0.82	5.84	0.22	0.28	0.16	12.43	3.29	1.82	17.27	0.018
Control ecosystem	0.57	6.47	0.48	0.32	0.2	2.55	2.23	8.93	85.22	0.005

ENA: ecological network analysis indicator. The abbreviations of D/H, TE, A/C, CI, SOI, FCI, FML, TPP/TR, TPP/TB, and TB/TST are the same as in Table 1.

**Table 7:** Evaluation results of the fuzzy synthetic evaluation for the status of marine ranching and control ecosystems.

Type	Ecosystem	Evaluation Grade of ENA Indices or Ecosystem Status				
		Poor	Relatively Poor	Medium	Relatively Good	Good
CI	Marine ranching ecosystem	0	0	0.67	0.33	0
	Control ecosystem	0	0	0	0.74	0.26
SOI	Marine ranching ecosystem	0	0.55	0.45	0	0
	control ecosystem	0	0	0.33	0.67	0
FCI	Marine ranching ecosystem	0	0	0	0.43	0.57
	control ecosystem	0.12	0.88	0	0	0
FML	Marine ranching ecosystem	0	0	0.12	0.88	0
	control ecosystem	0.09	0.91	0	0	0
D/H	Marine ranching ecosystem	0	0.32	0.68	0	0
	control ecosystem	0	0.4	0.6	0	0
A/C	Marine ranching ecosystem	0.42	0.58	0	0	0
	control ecosystem	0.03	0.97	0	0	0
TPP/TR	Marine ranching ecosystem	0	0	0	0.82	0.18
	control ecosystem	0.45	0.55	0	0	0
TPP/TB	Marine ranching ecosystem	0	0	0	0.99	0.01
	control ecosystem	0.44	0.56	0	0	0
TB/TST	Marine ranching ecosystem	0	0	0.13	0.88	0
	Control ecosystem	0.6	0.4	0	0	0
TE	Marine ranching ecosystem	0.25	0.75	0	0	0
	control ecosystem	0.09	0.91	0	0	0
Total ecosystem status	Marine ranching ecosystem	0.08	0.26	0.23	0.35	0.09
	Control ecosystem	0.14	0.54	0.11	0.17	0.03

The values in the table indicate the degree of membership for each indicator and the ecosystem within each evaluation grade. The highest value represents the status level of the indicators or ecosystem.

**Table 8:** Carrying capacity of the marine ranching and control ecosystems.

Functional Group	Carrying Capacity (t/km^2^)	
	Marine Ranching	Control Ecosystem
Pelagic fishes	2.12	0.48
Large and medium demersal fishes	0.23	0.24
Sparids	0.38	0.027
Leiognathidae	0.9	0.26
Small demersal fishes	0.62	0.16
Scorpaenidae	0.62	0.11
Gobiidae	0.87	0.098
Mantis shrimps	0.34	0.12
Large crabs	0.85	0.40
Other crabs	0.86	0.135
*Metapenaeopsis barbata*	1.6	0.40
Other shrimps	3.1	0.95
Cephalopods	0.34	0.12
Gastropods	1.82	0.36
barnacles	90	/
Oysters	96	/
Mussels	163	/

**Table 9:** The enhancing potential of each functional group in the marine ranch.

Category	Functional Group	Enhancing Potential (t/km^2^)
TL 3.0–3.5	Large and medium demersal fishes	0.05
	Sparids	0.05
	Small demersal fishes	0.44
	Scorpaenidae	0.60
	Gobiidae	0.80
	Cephalopods	0.27
	Mantis shrimps	0.17
	Other crabs	0.56
TL 2.5–3.0	Leiognathidae	0.52
	Pelagic fishes	2.01
	Large crabs	0.55
TL 2.0–2.5	Other shrimps	1.60
	*Metapenaeopsis barbata*	1.08
	Gastropods	0.94
	Barnacles	60.00
	Oysters	74.24
	Mussels	82.60
	Sea urchins	15.40

**Table 10:** ENA indicators in marine ranching under different simulation scenarios.

Simulation Scenario	ENA Indicators									
	Ecosystem Function			Food Web Structure				Ecosystem Maturity		
	D/H	TE	A/C	CI	SOI	FCI	FML	TPP/TR	TPP/TB	TB/TST
Only MOB	0.74	5.25	0.27	0.28	0.16	20.12	3.97	1.18	10.89	0.02
MOB + fishing	/	/	/	/	/	/	/	/	/	/
Only crab	0.73	5.14	0.22	0.28	0.15	12.45	3.3	1.82	17.24	0.02
Crab + fishing	/	/	/	/	/	/	/	/	/	/
Only fish	0.73	5.45	0.22	0.28	0.13	12.44	3.29	1.81	17.22	0.02
Fish + fishing	/	/	/	/	/	/	/	/	/	/
MOB + crab	0.73	5.22	0.27	0.28	0.16	20.13	3.97	1.19	10.9	0.02
MOB + crab + fishing	0.73	5.68	0.27	0.28	0.16	20.14	3.97	1.19	10.9	0.02
MOB + fish	0.73	5.48	0.27	0.28	0.14	20.11	3.97	1.18	10.87	0.02
MOB + fish + fishing	/	/	/	/	/	/	/	/	/	/
Crab + fish	0.74	5.46	0.22	0.28	0.12	12.39	3.29	1.81	17.21	0.02
Crab + fish + fishing	/	/	/	/	/	/	/	/	/	/
MOB + crab + fish	0.74	5.44	0.27	0.28	0.14	20.12	3.97	1.19	10.88	0.02
MOB + crab + fish + fishing	0.74	5.89	0.27	0.28	0.14	20.13	3.97	1.19	10.88	0.02
Only fishing	/	/	/	/	/	/	/	/	/	/

Crab and fish represent the functional groups of large crabs and scorpaenidae, respectively. MOB: mussels, oysters, and barnacles.

**Table 11:** Evaluation results of the fuzzy synthetic evaluation for the status of the marine ranch under different simulation scenarios.

Simulation Scenario	Poor	Relatively Poor	Medium	Relatively Good	Good
Only MOB	0.05	0.29	0.22	0.08	0.36
Only crab	0.10	0.26	0.20	0.29	0.15
Only fish	0.10	0.29	0.17	0.34	0.10
MOB + crab	0.05	0.28	0.16	0.08	0.36
MOB + crab + fishing	0.03	0.29	0.23	0.08	0.36
MOB + fish	0.04	0.34	0.17	0.08	0.36
Crab + fish	0.10	0.28	0.17	0.34	0.10
MOB + crab + fish	0.04	0.34	0.17	0.08	0.36
MOB + crab + fish + fishing	0.03	0.35	0.17	0.08	0.36

The values in the table indicate the degree of membership for the ecosystem within each evaluation grade. The highest value represents the status level of the ecosystem. MOB: mussels, oysters, and barnacles.

## Data Availability

The original contributions presented in the study are included in the article; further inquiries can be directed to the corresponding authors.

## Appendix A. Introduction of Ecopath Model

The Ecopath model is commonly used to quantitatively estimate mass transfer and energy flow in an ecosystem [62,78]. According to thermodynamic theories, the energy input and output of a certain biological functional group should be balanced, with productivity equaling the sum of mortalities (Equation (A1)). Another equation was used to determine the inner mass balance of the group (Equation (A2)), where the consumption (*Q*) of each group was equal to the sum of the production (*P*), respiration (*R_i_*), and unassimilated food (*U_i_*) of the group. The equations are as follows:

(A1) $$B_{i}\times {\left(\frac{P}{B}\right)}_{i}\times EE_{i\;}=\sum_{j}B_{j}\times {\left(\frac{Q}{B}\right)}_{j}\times DC_{ij}+Y_{i}+BA_{i}+E_{i\;\;\;\;\;\;\;\;\;\;\;\;\;\;\;\;\;\;}$$

(A2) $$B_{i}\times {\left(\frac{Q}{B}\right)}_{i}=B_{i}\times {\left(\frac{P}{B}\right)}_{i}+R_{i}+U_{i}$$

where *B_i_* is the biomass of functional group *i*, *(P/B)_i_* is the production ratio, *(Q/B)_i_* is the consumption ratio, *EE_i_* is the ecotrophic efficiency (the proportion of production utilized in the system), *DC_ij_* is the proportion of prey *i* in the diet of predator *j*, *Y_i_* is the fishery mortality, *BA_i_* is the biomass accumulation rate, and *E_i_* is the net migration rate. In the parameter input process, *B_i_*, *(P/B)_i_*, *(Q/B)_i_*, and *DC_ij_* are required. If one of the first three values is missing, the corresponding EE value can be designated. In addition, *E_i_* and catches are also required.

## Appendix B. Introduction of the Ecosim Model

Ecosim is a time-scale based dynamic model based on Ecopath [62]. It incorporates a time-dynamic aspect by varying the Ecopath model over time increments, simulating systemic response changes over the time series. The equation used is as follows:

(A3) $$\frac{dB_{i}}{d_{t}}=g_{i}\Sigma Q_{ji}-\Sigma Q_{ij}+I_{i}-\left(F_{i}+Mo_{i}+e_{i}\right)\;B_{i}$$

where *dB_i_/dt* is the rate of change in the biomass, *g_i_* is the net growth efficiency, *Q_ji_* is the consumption rate of function group *j* to function group *i*, *I_i_* is the emigration rate, *F_i_* is the fishing mortality rate, *M_oi_* is the non-predatory natural mortality, *e_i_* is the move-in rate, and *B_i_* is the biomass of functional group *i* [62].

The Ecosim models use foraging arena theory’s, where each predator/prey interaction is defined by vulnerability parameters that affect the predator consumption rate (Equation (A4)) to describe the top-down and bottom-up controls of the predator/prey interactions. Vulnerability parameters can range between 1 and infinity, with 2 as the default. For each predator-prey interaction consumption rates, *Q_ij_*, are calculated as:

(A4) $$Q_{ij}=\frac{a_{ij}\times v_{ij}\times B_{i}\times P_{j}\times T_{i}\times T_{j}\times \left(M_{ij}/D_{j}\right)}{v_{ij}+v_{ij}\times T_{i}\times M_{ij}+a_{ij}\times M_{ij}\times P_{i}\times \left(T_{j}/D_{j}\right)}\times f\left(Env_{function},t\right)$$

where *a_ij_* is the effective search rate for predator *j* feeding on a prey *i*, *v_ij_* is vulnerability expressing the rate with which prey *i* move between being vulnerable and non-vulnerable, B is prey biomass, *P_j_* is predator biomass (or abundance for split groups), *T_i_* represents prey relative feeding time, *T_j_* is predator relative feeding time, *M_ij_* are the mediation forcing effects, and *D_j_* represents handling time as a limit to consumption rate [149,150]. *f_(Env_function, t)_* is the environmental response function that restricts the size of the foraging arena to account for external environmental drivers changing over time [149].

## Appendix C. Introduction of Ecological Network Analysis Indicators

D/H reflects the ratio of the transfer of flows (carbon, energy, or nutrients) from detritus or autotrophs to trophic level II in a food web [52]. A high D/H value indicates a shift to a more detritus-based food web, which indicates more maturity, stability, and a more resilient ecosystem.

TE is derived from the logarithmic mean of the efficiencies of the trophic levels between II and IV. A high TE value is characteristic of a mature ecosystem or of an oligotrophic ecosystem with scarce elements. Various disturbances such as species invasions and eutrophication would lower the TE value [52].

The ratio of ascendency or overhead to system throughput is derived from information theory as a measure of the average mutual information in a system and could also be a measurement of ecosystem growth and development. Relative Ascendency (A/C) is an index of the organization of a food web and concerns the efficiency of an organization. A/C is higher when pathways are few in number and transport most of the material. The highest theoretical value of ascendency can be achieved when all system components only have a single input and output [110]. The opposite of A/C is the relative overhead (O/C) for two values that add up to 1. The latter represents redundant flows, which are additional (parallel) pathways between nodes [151]. As a high A/C indicates high efficiency and a high O/C indicates high resilience, a tradeoff or balance between A/C and O/C (efficiency vs. resilience) exists, which leads to the interpretation that a good ecosystem consists of different types of pathways, some of which are efficient while others are redundant. Since the O/C and A/C are mutually exclusive, only A/C is selected as an indicator.

The CI and SOI are important indices that are used to describe food web features. High values of the CI and SOI could reflect a high diversity of diet composition, while low values indicate a linear food web pattern rather than a web-like structure. The FCI represents the fraction of an ecosystem throughput that is recycled compared with total throughput. It is assumed that mature systems show a higher degree of recycling than immature systems [152]. A high cycling index increases a system’s maturity and stability. The higher the cycling index, the more the ecosystem is released from stress. APL measures the mean number of groups that a unit of flux will experience from its entry into the system until it leaves the system. As the diversities of flows and cycling are expected to increase with maturity, it is assumed that the FML will be higher in a more mature system.

The TPP/TR and TPP/TB ratios are both significant parameters that reflect the maturity of an ecosystem. When an ecosystem develops into maturity, the TPP/TR ratio approaches 1, and the TPP/TB ratio decreases. In ecosystems suffering from organic pollution, this ratio of TPP/TR is expected to be less than 1 [62,120]. The TB/TST value is directly proportional to system maturity. This ratio tends to be low in developing ecosystems and increases with the maturity and stability of the system [78].

## Appendix D. Data Sources for the Ecopath Models of the Marine Ranching and Control Ecosystems

The data sources for the B, P/B, Q/B, and diet composition of each group in the two ecosystems are described in Table A3.

## Appendix E. Tables

**Table A1:** Trophic groups and main species included in the marine ranching ecosystem Ecopath model.

Numbers	Functional Groups	Maine Species
1	Pelagic fishes	*Konosirus punctatus*, *Thryssa dussumieri*, *Trachinotus ovatus*
2	Large and medium demersal fishes	*Sillago sihama*, *Alepes djedaba*, *Scatophagus argus*, *Takifugu alboplumbeus*, *Saurida elongate*, *Lagocephalus spadiceus*, *Ilisha melastoma*, *Ilisha elongate*, *Planiliza affinis*
3	Sparids	*Plectorhinchus lineatus*, *Acanthopagrus latus*, *Evynnis cardinalis*, *Jaydia lineata*, *Acanthopagrus schlegelii*
4	Leiognathidae	*Equulites rivulatus*, *Leiognathus brevirostris*, *Leiognathus berbis*
5	Small demersal fishes	*Pennahia anea*, *Psenopsis anomala*, *Callionymus curvicornis*, *Solea ovata*, *Johnius belangerii*, *Johnius fasciatus*, *Osteomugil strongylocephalus*, *Sardinella albella*, *Pennahia macrocephalus*
6	Scorpaenidae	*Vespicula trachinoides*, *Sebastiscus marmoratus*
7	Gobiidae	*Cryptocentrus russus*, *Parachaeturichthys polynema*, *Amoya caninus*, *Tridentiger obscurus*, *Myersina filifer*
8	Mantis shrimps	*Oratosquilla oratoria*
9	Large crabs	*Charybdis japonica*, *Portunus trituberculatus*, *Portunus pelagicus*
10	Other crabs	*Pilumnopeus eucratoides*, *Charybdis helleri*, *Parthenope Validus*, *Charybdis acuta*, *Thalamita sima*, *Dorippe facchino*, *Halimede ochtodes*
11	*Metapenaeopsis barbata*	*M. barbata*
12	Other shrimps	*Alpheus hoplocheles*, *Marsupenaeus japonicus*, *Trachypenaeus curvirostris*, *Metapenaeus intermedius*, *Parapenaeopsis hungerfordi Alcock*
13	Cephalopods	*Loliolus japonica*
14	Sea urchins	*Anthocidaris crassispina*, *Hemicentrotus pulcherrimus*
15	Gastropods	*Patelloida pygmaea*, *Nassarius semiplicatus*, *Turritella terebra bacillum*, *Cellana toreuma*, *Murex trapa*
16	Barnacle	*Amphibalanus reticulatus*
17	Oysters	*Crassostrea gigas*, *Ostrea denselamellosa*
18	Mussels	*Perna viridis*
19	Other bivalves	*Dosinia aspera*, *Timoclea scabra*, *Vepricadium coronatum*, *Lucina scarlatoi*, *Clausinella isabelline*, *Ruditapes variegatus*, *Lutraria sieboldii*
20	Other benthos	*Anthopleura xanthogrammica*, *Stichopus variegatus*
21	Zooplankton	*Paracalanus parvus*, *Parvocalanus carssirostris*, *Oithona nana*, *Corycaeus dahli*, *Corycaeus affinis*
22	Phytoplankton	*Chaetoceros lorenzianus*, *Eucampia cornuta*, *Eucampia zoodiacus*, *Stephanopyxis palmeriana*, *Chaetoceros constrictus*
23	Detritus	Detritus in water, detritus in sediment

**Table A2:** Trophic groups and main species included in the control ecosystem Ecopath model.

Number	Functional Group	Maine Species
1	Pelagic fishes	*Konosirus punctatus*
2	Large and medium demersal fishes	*Sillago sihama*, *Scatophagus argus*, *Trichiurus japonicus*, *Nematalosa japonica*, *Atule mate*, *Lagocephalus spadiceus*, *Ilisha elongata*
3	Sparids	*Lutjanus erythropterus*, *Evynnis cardinalis*
4	Leiognathidae	*Leiognathus brevirostris*, *Equulites rivulatus*
5	Small demersal fishes	*Pennahia anea*, *Psenopsis anomala*, *Callionymus curvicornis*, *Johnius belangerii*, *Johnius fasciatus*, *Osteomugil strongylocephalus*
6	Scorpaenidae	*Sebastiscus marmoratus*
7	Gobiidae	*Cryptocentrus russus*, *Trypauchen vagina*, *Odontamblyopus lacepedii*, *Parachaeturichthys polynema*, *Amoya caninus*, *Myersina filifer*
8	Mantis shrimps	*Oratosquilla oratoria*
9	Large crabs	*Portunus pelagicus*
10	Other crabs	*Charybdis hellerii*, *Charybdis acuta*, *Thalamita sima*, *Pilumnopeus eucratoides*
11	*Metapenaeopsis barbata*	*Metapenaeopsis barbata*
12	Other shrimps	*Marsupenaeus japonicus*, *Trachypenaeus curvirostris*
13	Cephalopods	*Loliolus japonica*
14	Gastropods	*Architectonica perspectiva*
15	Bivalves	*Vepricadium coronatum*, *Trapezium sublaevigatum*, *Scapharca anomala*
16	Other benthos	*Phascolosoma esculenta*
17	Zooplankton	*Paracalanus parvus*, *Parvocalanus carssirostris*, *Paracalanus nanus*, *Corycaeus dahli*, *Oithona attenuata*
18	Phytoplankton	*Chaetoceros constrictus*, *Thalassionema nitzschioides*, *Odontella sinensis*, *Bacteriastrum furcatum Shadbolt*, *Thalassionema nitzschioides*
19	Detritus	Detritus in water, detritus in sediment

**Table A3:** Data sources for the B, P/B, Q/B, and diet composition of each group in the marine ranching and control ecosystems.

Functional Group	B	PB	QB	Diets
Pelagic fishes	By trawl nets in non-reef areas and SCUBA videos in reef areas. Trawl nets near reefs were also conducted for correction of the video data	Estimated according to empirical formula [153]	Estimated according to empirical formula [154]	[155,156]
Large and medium demersal fishes	Same as pelagic fishes	Estimated according to empirical formula [153]	Estimated according to empirical formula [154]	[157,158,159,160]
Sparids	Same as pelagic fishes	Estimated according to empirical formula [153]	Estimated according to empirical formula [154]	[161,162]
Leiognathidae	Same as pelagic fishes	Estimated according to empirical formula [153]	Estimated according to empirical formula [154]	[163]
Small demersal fishes	Same as pelagic fishes	Estimated according to empirical formula [153]	Estimated according to empirical formula [154]	[21,148,164,165]
Scorpaenidae	Same as pelagic fishes	Estimated according to empirical formula [153]	Estimated according to empirical formula [154]	[166]
Gobiidae	Same as pelagic fishes	Estimated according to empirical formula [153]	Estimated according to empirical formula [154]	[167,168,169]
Mantis shrimps	By trawl nets in non-reef areas and cage nets in reef areas [37]. Trawl nets and cage nets near reefs were also conducted for correction of cage nets data	Estimated according to empirical formula [170]	By measuring the R/B first [171] and calculating the Q/B according to the following: Q/B = P/B + R/B + U/B; U = 0.2 Q [64]; U/B = 0.2 Q/B	[172]
Large crabs	Same as mantis shrimps	Estimated according to empirical formula [170]	Same as mantis shrimps	[173]
Other crabs	Same as mantis shrimps	Estimated according to Empirical formula [170]	Same as mantis shrimps	[174]
*Metapenaeopsis barbata*	Same as mantis shrimps	Estimated according to Empirical formula [170]	Same as mantis shrimps	[175]
Other shrimps	Same as mantis shrimps	Estimated according to Empirical formula [170]	Same as mantis shrimps	[176]
Cephalopods	Same as mantis shrimps	Estimated according to Empirical formula [170]	Same as mantis shrimps	[177]
Sea urchins	By SCUBA grasping with a 0.5 × 0.5 m quadrats	Estimated according to Empirical formula [170]	Same as mantis shrimps	[178]
Gastropods	Same as sea urchins	Estimated according to Empirical formula [170]	Same as mantis shrimps	[179]
Barnacle	By SCUBA grasping with a 0.5 × 0.5 m quadrats, and samples of oysters were collected using a small knife	Estimated according to Empirical formula [170]	Same as mantis shrimps	[180]
Oysters	Same as barnacle	Estimated according to Empirical formula [170]	Same as mantis shrimps	[37]
Mussels	Same as barnacle	Estimated according to Empirical formula [170]	Same as mantis shrimps	[37]
Other bivalves	By collecting the samples with a sediment sampler	Estimated according to Empirical formula [170]	Same as mantis shrimps	[181]
Other benthos	By collecting the samples with a sediment sampler	Estimated according to Empirical formula [170]	By measuring the R/B first [171] and calculating the Q/B according to the following: Q/B = P/B + R/B + U/B; U = 0.35 Q [64]; U/B = 0.35 Q/B	[37]
Zooplankton	By vertical towing using plankton nets	[182]	Obtained from Duan et al. (2009) [182]	
Phytoplankton	Calculated according to Chl a [58]	[183]		
Detritus	Estimated according to empirical formula [99]			

B: biomass; P/B: production/biomass; Q/B: consumption/biomass.

**Table A4:** Diet matrix of the marine ranching ecosystem.

Numbers	Prey\Predator	1	2	3	4	5	6	7	8	9	10	11	12	13	14	15	16	17	18	19	20	21
1	Pelagic fishes		0.02																			
2	Large and medium demersal fishes		0.05																			
3	Sparids		0.01																			
4	Leiognathidae																					
5	Small demersal fishes		0.02	0.023		0.02	0.03							0.02								
6	Scorpaenidae			0.001																		
7	Gobiidae		0.015	0.02			0.01	0.01														
8	Mantis shrimps					0.04	0.01															
9	Large crabs			0.02			0.02		0.01													
10	Other crabs	0.005	0.03	0.06	0.04	0.07	0.1	0.05	0.079	0.03	0.02			0.1								
11	*Metapenaeopsis barbata*		0	0.081	0.05	0.05	0.05	0.05	0.03	0.02	0.02			0.1								
12	Other shrimps	0.03	0.21	0.464	0.08	0.38	0.15	0.15	0.096	0.04	0.04	0.01		0.13							0.03	
13	Cephalopods		0.03	0.02							0.002			0.03								
14	Sea urchins								0.1													
15	Gastropods								0.05		0.053	0.05	0.05									
16	Barnacle			0.096					0							0.05						
17	Oysters								0	0.170	0.140					0.1					0.04	
18	Mussels				0.09	0.11	0.17	0.17	0.16	0.544	0.561	0.1	0.04	0.04		0.07					0.03	
19	Other bivalves				0.28	0.13	0.31	0.27	0.215	0.013	0.014	0.05	0.07	0.17		0.05					0.03	
20	Other benthos							0.2			0.13					0.1						
21	Zooplankton	0.648	0.1	0.055	0.22		0.15	0.1	0.16		0.02	0.13	0.13	0.31	0.1	0.05	0.02	0.02	0.02	0.02	0.2	
22	Phytoplankton	0.317			0.14										0.1		0.35	0.65	0.637	0.6		0.66
23	Detritus				0.1	0.1			0.1	0.184		0.66	0.66		0.8	0.58	0.63	0.33	0.343	0.38	0.67	0.34
24	Import		0.515	0.1		0.1						0	0.05	0.1								
25	Sum	1	1	1	1	1	1	1	1	1	1	1	1	1	1	1	1	1	1	1	1	1

**Table A5:** Diet matrix of the control ecosystem.

Number	Prey\Predator	1	2	3	4	5	6	7	8	9	10	11	12	13	14	15	16	17
1	Pelagic fishes		0.008															
2	Large and medium demersal fishes		0.05															
3	Sparids																	
4	Leiognathidae			0.04														
5	Small demersal fishes		0.02	0.063		0.03	0.02							0.02				
6	Scorpaenidae			0.001														
7	Gobiidae		0.015	0.092			0.06	0.01										
8	Mantis shrimps					0.04	0.01											
9	Large crabs			0.02			0.02		0.01					0.08				
10	Other crabs	0.005	0.03	0.16	0.02	0.03	0.1	0.05	0.015	0.01	0.02			0.02				
11	*Metapenaeopsis barbata*			0.17	0.02	0.02	0.1	0.02	0.03	0.0084	0.02			0.02				
12	Other shrimps	0.03	0.21	0.198	0.12	0.38	0.1	0.18	0.21	0.072	0.04	0.01		0.21				
13	Cephalopods		0.02	0.03							0.008			0.03				
14	Gastropods										0.047	0.05	0.05					
15	Bivalves			0.096	0.36	0.3	0.44	0.44	0.375	0.7	0.71	0.1	0.11	0.21	0.365		0.01	
16	Other benthos							0.2			0.135				0.005			
17	Zooplankton	0.648	0.1	0.03	0.24		0.15	0.1	0.26		0.02	0.18	0.13	0.31	0.05	0.01	0.067	
18	Phytoplankton	0.317			0.14											0.6		0.66
19	Detritus				0.1	0.1			0.1	0.21		0.66	0.71		0.58	0.39	0.923	0.34
20	Import		0.547	0.1		0.1								0.1				
21	Sum	1	1	1	1	1	1	1	1	1.0004	1	1	1	1	1	1	1	1

**Table A6:** Values of the indicators of an ecological network analysis of coastal ecosystems worldwide.

	Study Area	Time	Ecosystem Function			Food Web Structure				Ecosystem Maturity			**Data Sources**
			D/H	A/C	TE	CI	SOI	FCI	FML	TPP/TR	TPP/TB	TB/TST	
1	Tongoy Bay	1992		0.396	6.420	0.320	0.090	1.500	3.340	3.500	26.600	0.016	[18,33,35,37,56,105,121,130,148,165,182,184,185,186,187,188,189,190,191,192,193,194,195,196,197,198,199,200,201,202,203,204,205,206,207,208,209,210,211,212,213,214,215,216,217,218,219,220,221,222,223,224,225,226,227,228,229,230,231,232,233,234,235,236,237,238,239,240,241,242,243,244,245,246,247,248,249,250,251]
2		2002		0.318	7.130	0.320	0.110	2.900	3.490	2.500	12.200	0.034	
3		2012		0.339	9.410	0.310	0.090	2.200	3.340	2.400	16.000	0.026	
4	Pagasitikos Gulf	2008					0.250			1.470	9.100	0.030	
5	North and Central Gulf of California	1980s	1.652	0.347	22.200	0.133	0.320	6.240		2.500	16.500	0.022	
6	The southeastern Gulf of California	1994–1997	0.322	0.290	14.500	0.200	0.200	5.200		2.344	1.947	0.039	
7		2006–2007	0.233	0.340	7.500	0.100	0.100	4.700		12.192	13.699	0.034	
8	Bay of Seine and eastern part of the English Channel	2007–2013	0.520				0.173	9.160				0.030	
9	Seine estuary	1996–2002			4.500		0.180	8.520	2.820	2.590	48.680	0.007	
10	Seine estuary	1996–2002			5.200		0.190	18.940	4.010	1.220	8.990	0.022	
11	Seine estuary	1996–2002			6.800		0.160	3.650	2.630	1.090	6.950	0.009	
12	Seine estuary	1996–2002			9.100		0.190	13.860	3.600	1.560	12.020	0.020	
13	Seine estuary	1996–2002			7.400		0.180	11.230	3.260	1.930	16.680	0.016	
14	Seine estuary	1996–2002			9.900		0.160	20.650	4.330	1.110	9.150	0.020	
15	Black Sea	1960–1969			4.275		0.070	9.400	2.660	1.630	132.000	0.003	
16	Black Sea	1980–1987			4.800		0.120	4.600	2.480	2.270	90.960	0.004	
17	Black Sea	1988–1994			5.500		0.120	2.760	2.320	3.610	116.770	0.004	
18	Black Sea	1995–2000			3.675		0.120	15.010	2.940	1.160	89.850	0.004	
19	Northwestem Mediteranean sea	1999–2003			14.300		0.190	9.120	2.750	4.890	32.000		
20	Gulf of Lions	2000–2009			19.700		0.210	11.870	3.990	2.090	15.100		
21	Lower continental slope of the Catalan sea	2009			15.700		0.290	4.200					
22	Northcentral Adriatic Sea	1990			10.000		0.190	14.700	5.410	2.730	8.820		
23	North Aegean Sea	2003–2006			17.400		0.180	14.600	3.630	2.990	16.210		
24	Greek Ionian Sea	1998–2006			13.100		0.360	14.330	5.850	1.830	23.250		
25	Gulf of Gabes	2000–2005			19.240			7.350	3.050	2.410	16.750		
26	Gulf of Cadiz	2009			14.900		0.180	3.000	2.430	3.300	39.800		
27	Jade Bay (German Wadden Sea)	1935–1937	1.800	0.349	4.960	0.432			6.100		5.130		
28		1975–1977	1.300	0.389	2.920	0.289			4.260		3.700		
29		2009	4.200	0.419	4.220	0.310			7.690		3.940		
30	Mejillones Bay	2005–2012		0.241		0.200	0.074	4.880	2.920	1.550	6.280	0.060	
31	Antofagasta Bay	2005–2012		0.285		0.180	0.069	6.020	2.770	1.400	7.190	0.050	
32	Nigerian coastal waters	1985			12.780	0.284	0.273	2.238	9.230				
33		2000			11.930	0.284	0.256	2.133	9.390				
34	Somme Bay	1998		0.350	4.000	0.250	0.009	12.200		15.509	21.816	0.012	
35	Kuosheng Bay	1998–2001	2.444		6.500	0.480	0.520	32.000	4.400	1.060	40.000	0.006	
36	Cadiz Gulf	2009	0.283		14.900	0.250	0.180	3.000	2.430	3.300	39.800	0.010	
37	Estuary of Sirinhaém River in northeastern Brazil	2013–2014	0.980	0.290	11.580	0.270	0.160	5.610		2.590	32.590	0.010	
38	Poonthura Estuary	2016–2020	0.240	0.150	12.450	0.350	0.380	17.940		0.460	5.210	0.020	
39	Estuarine ecosystem around bight of Benin, Nigeria		0.461	0.423	6.800	0.327	0.288	1.700	2.300	6.325	82.615	0.005	
40	South Catalan Sea	late 1970	1.004	0.417	11.500		0.220	4.980	2.400	6.840	33.960	0.010	
41	South Catalan Sea	mid 1990	0.670	0.358	12.200		0.220	5.770	2.560	4.820	26.740	0.010	
42	South Catalan Sea	early 2000	0.664	0.411	13.300		0.200	6.220	2.410	6.710	28.980	0.010	
43	Gulf of Maine	1980				0.265	0.307	0.650	2.010	2.090	42.560	0.011	
44	Gulf of Maine	1990				0.265	0.290	3.630	2.310	1.760	18.940	0.023	
45	A marine protected area on the coast of Sénégal	2003		0.255		0.340	0.146	3.540	2.500	1.910	18.290	0.021	
46		2006–2008		0.272		0.340	0.154	3.660	2.500	2.080	18.220	0.022	
47	Venezuela Shelf Ecosystem	1986–1989		0.399	6.600		0.135	2.200	4.050		27.000	0.023	
48	Eastern Central Pacific Ocean	1986–1989						2.000	2.400				
49	Gulf of Mexico	1986–1989		0.391			0.195	2.100	3.030		7.000	0.015	
50	British Columbia Shelf	1991–2007		0.401			0.140		2.030		21.100	0.180	
51	Northern Benguela Upwelling ecosystem	1991–2007		48.500				4.220	3.500				
52	Terminos Lagoon, Mexico	1980–1988						7.000	10.000				
53	Sandy Barrier Lagoon, Taiwan	1997						10.800	3.380				
54	Boca Paila Reef, Mexico	1990–1998	0.860					13.400		1285.000	15.600		
55	Channel of São Sebastião ecosystems	1990–1997		25.400		0.260	0.210	30.100		0.700	11.200	0.012	
56	Inner shelf of São Sebastião ecosystems	1990–1997		23.200		0.280	0.210	25.800		1.900	30.100	0.010	
57	Bengal Bay	2003		0.387	5.900	0.420	0.220	10.000	2.580	1.350	14.690	0.026	
58	Northern and Central Adriatic Sea	1990s.	1.680	27.000	10.000		0.190	14.700	3.340	2.730	8.800	0.030	
59	Prince Edward Islands marine ecosystem	1960	0.300		11.100	0.204	0.220			1.560	30.380	0.012	
60	Prince Edward Islands marine ecosystem	1980	0.300		11.000	0.204	0.210			1.560	30.390	0.012	
61	Prince Edward Islands marine ecosystem	2000	0.300		11.000	0.204	0.200			1.560	30.440	0.012	
62	Kerguelen Island marine ecosystem	2005				0.230	0.170			1.160	12.980	0.024	
63	South Georgia	2012				0.190	0.410			0.890	6.820	0.031	
64	South Shetlands	2003				0.250	0.160			2.750	53.090	0.008	
65	Falklands marine ecosystem	2005				0.180	0.280			12.310	83.950	0.006	
66	Antarctic Peninsula	2005				0.270	0.160			5.140	9.460	0.048	
67		2008				0.200	0.150			10.350	11.480	0.037	
68		2012				0.200	0.180			1.580	16.610	0.021	
69	Southern Plateau, New Zealand	1989–1996				0.160	0.290			1.490	48.560	0.006	
70	Jurien Bay, Western Australia	2005–2006			9.600	0.160	0.250			1.100	2.100	0.080	
71	Eritrean Red Sea	1997–2005			8.600	0.460	0.210	10.760	3.640	1.100	11.950	0.023	
72	Subtidal area in Tongoy Bay, Chile	1971–2001			11.500	0.200	0.140	2.610	2.400	2.700	12.220	0.034	
73	Western Scotland coast ecosystem	1997–2003				0.290	0.180	2.540	2.060	4.510	30.610	0.013	
74	Rocky coastal ecosystem Bahia Tortugas, Mexico	2006–2008		0.200		0.230	0.230			1.050	1.340		
75	Sublittoral community of the Bay of Calvi, Corsica	1983–1998			11.300		0.340	21.690	4.260	0.800	1.500	0.095	
76	Eastern Bering Sea ecosystem	1950s		0.325		0.290	0.183	13.200	3.470	0.940	5.850	0.046	
77		1980s		0.309		0.300	0.157	11.100	3.510	0.780	4.940	0.045	
78	West Coast of Sabah, Malaysia	1972				0.270	0.220			2.070	19.620	0.020	
79	West Coast of Sarawak, Malaysia	1972				0.270	0.220			2.080	19.370	0.020	
80	San Pedro Bay, Leyte, Philippines					0.450	0.290			1.390	46.810	0.008	
81	Karnataka Arabian Sea	1999–2001		0.329	13.400	0.382	0.299	6.030	2.810	1.283	29.900	0.012	
82	northern Benguela upwelling system, Namibia	1990–1995		0.485		0.194	0.252	4.220	3.500				
83	Tenerife and La Gomera Islands marine ecosystem	2016		0.268	18.930	0.190	0.280	14.440	3.490	1.980	7.790	0.040	
84	Pearl River Delta coastal sea ecosystem	1997–1999				0.237	0.327			2.867	18.134	0.017	
85	Wangjiadao Islands marine ecosystem	2019	0.633		49.100	0.240	0.180	13.890	3.550			1.650	
86	Gulf of Ulloa	1980–2006		0.650	46.000	0.200	0.150	0.160				33.000	
87	Isla del Coco, Costa Rica, Eastern Tropical Pacific	2015	1.383		2.320	0.170	0.400	6.500		0.248	0.380		
88	Northern Hangzhou Bay	2006–2007		0.310	8.900	0.310	0.350	25.000	2.170	2.560	69.250	0.005	
89	Beibu Gulf	1959–1961	0.750	0.579	7.100	0.316	0.186	1.860	1.755	1.013	28.920	0.062	
90		1990s	0.460	0.476	9.400	0.310	0.171	0.840	1.206	2.184	54.355	0.010	
91		1997–1999			12.200	0.333	0.319	0.840	1.206	3.182	24.547	0.018	
92	Bohai Sea	1982			12.300	0.350				9.745	127.493	0.004	
93		1992–1993			16.200		0.341			8.400	86.043	0.006	
94		2014–2015			5.100	0.330	0.140			5.380	99.830	0.005	
95		2016	0.367	0.626	11.350	0.341	0.276	0.892	2.091	11.713	168.789	0.003	
96	Xiangyun Bay	2019–2020	2.036	0.268	9.160	0.240	0.161	19.810	4.071	0.748	4.257	0.038	
97		2019–2020	1.240	0.319	7.570	0.247	0.130	11.810	2.883	2.657	30.734		
98	Northern South China Sea	1989–1992						17.500	4.537	2.352	6.000	0.060	
99		1997–2000						8.380	2.790	2.818	40.252	0.010	
100		2000–2004						2.630	2.302	8.676	60.839	0.008	
101		2007–2008			11.500	0.290	0.239	4.380	2.476	2.596	25.000	0.016	
102		2015–2016			21.940	0.313	0.325	13.680	3.775	1.005	32.190	0.008	
103	Southern East China Sea	1999–2002	0.637		12.000	0.330	0.213	4.100	2.398	3.060			
104	East China Sea	1997–2000	0.507		14.600	0.190	0.201	0.180	1.903	3.383	43.458		
105	South Yellow Sea	2000–2001	0.583	0.248	8.100	0.360	0.210	9.830		1.430	41.270	0.028	
106	Southwest Yellow Sea	2006–2009	0.618		13.220	0.280	0.217	3.983	2.444	2.541	50.362	0.008	
107	Yangtze River Estuary	1985–1986	0.723		12.400	0.471	0.103	9.350	2.778	1.724	31.483	0.011	
108		2000			9.400	0.449	0.256		2.215	5.293	79.021	0.006	
109		2006			9.900	0.414	0.313	0.060	2.595	1.815	41.672	0.009	
110		2004	0.685		14.700	0.539	0.069	4.200	2.461	2.527	50.350	0.008	
111		2012	0.544		9.400	0.371	0.196	5.990	2.500	2.095	67.525	0.006	
112		2016–2017	0.073		9.300	0.345	0.321			1.245	53.402	0.007	
113		2020	0.451		9.850	0.388	0.234			3.200	31.910	0.010	
114	Haizhou Bay	2003			13.800	0.270	0.210	0.030	2.220	4.500	40.339	0.012	
115		2013			7.900	0.415	0.174	0.114	2.301	1.331	44.986	0.009	
116		2013		0.647	5.620				3.093	4.720	92.404	0.005	
117		2015		0.488	5.660			0.184		1.299	44.000	0.007	
118		2018			12.630	0.429	0.204	1.392		7.069	56.866	0.017	
119	Daya Bay	2010–2011	0.525	0.363	10.900	0.249	0.138	2.170	2.210	3.500	82.500	0.005	
120	Laizhou Bay	2009–2010			6.200	0.290	0.170	0.070		1.530	24.540	0.014	
121	Jiaozhou Bay	2011	0.341		14.400	0.310	0.160	2.470	2.300	3.180	30.040	0.010	
122		2015–2016			16.350	0.248	0.116	4.269	2.436	2.518	32.873	0.012	
123	Yellow River Estuary	2012–2013	1.163		9.700	0.300	0.150	6.160		2.470	33.300	0.012	
124		2013–2014			5.400	0.380	0.120	2.800		3.450	38.910	0.011	
125	Artificial reef ecosystem near Yantai coast	2019			10.560	0.300	0.200			1.930			
126	Artificial reef ecosystem near Li Island	2014			11.700	0.320	0.140	5.460	2.690	1.820	6.600	0.060	
127	Artificial reef ecosystem in Laizhou Bay	2010–2012			12.800	0.440	0.360	12.230	3.300	1.035	15.580	0.019	
128	Artificial reef ecosystem in Laoshan Bay	2014–2016			10.800	0.290	0.330	20.950	4.000	1.130	13.610		
129	Gouqi Island marine ecosystem, Shanghai	2007–2008			12.700	0.330	0.220		2.950	1.250			
130	Artificial reef ecosystem west of Furong Island	2019–2020	0.620	0.320	8.920	0.160	0.120	22.670	5.170	1.010	9.620	0.020	
131	Artificial reef ecosystem in Xiangyun Bay	2017–2018	0.630	0.249	6.940	0.240	0.150	20.510	4.310	0.700	4.220	0.034	
132	Oyster–macroalgae reef ecosystem in Xiangyun Bay	2017–2018	0.610	0.266	9.120	0.240	0.160	19.710	4.070	0.750	4.257	0.038	
133	Artificial reef ecosystem near Dachen Island	2019–2020	0.570	0.278	12.460	0.240	0.270	19.810	4.000	1.300	32.160	0.008	
134	Artificial reef ecosystem near Wuzhizhou island	2019–2020	0.390	0.343	12.070	0.200	0.240	16.860	5.200	2.020	33.740	0.011	
135	Galapagos subtidal rocky reef ecosystem	1997–2003				0.250	0.160			0.480	5.060	0.030	
136	Qilianyu Islands coral reef ecosystem	2019	0.552		26.300	0.330	0.210	3.640	2.470	0.277		3.790	
137	Nanwan Bay coral reef ecosystem	2001–2003	1.400		9.900			3.500				4.400	
138	Tampalam Reefs ecosystem, Mexico	1990–1998	1.000					13.500		1.240	14.080		
139	Mahahual Reefs ecosystem, Mexico	1990–1998	1.000					15.400		1.270	12.450		

## References

[1] Kang X., Zhang H. (2010). Research progress on the evaluation of offshore marine ecosystem service functions and their values. Ocean. Dev. Manag..

[2] Qiu W. (2019). Influence of International Non-Governmental Organizations on the Rule of the International Law on Marine Environmental Protection: Taking an Example of IUCN. Master’s Thesis.

[3] Field J., Hempel G., Summerhayes C., Scientific Committee on Oceanic Research, International Council of Scientific Unions, Scientific Committee on Problems of the Environment (2002). International Council of Scientific Unions Oceans 2020: Science, Trends, and the Challenge of Sustainability.

[4] Sun X.X., Yu R.C., Hu Z.Y. (2016). Ecological security of coastal ocean and future marine ecosystem management strategies. Bull. Chin. Acad. Sci..

[5] Yang Z.Y., Zhang D. (2023). The high-quality development of Chinese fisheries from the perspective of the all-encompassing approach to food: Current situation, problems and improvement strategies. Chin. Fish. Econ..

[6] Sun R.Y., Li B., Zhuge Y., Shang Y.C. (1993). General Ecology.

[7] Lin C., Yang H., Chen Y., Jin X., Chen B., Li W., Ren Z., Leng S., Ding D. (2021). Construction and Development of Modern Marine Ranching Academic Review of the 230th Shuangqing Forum. Bull. Natl. Nat. Sci. Found. China.

[8] CCSC (2021). Outline of the 14th Five-Year Plan (2021–2025) for National Economic and Social Development and Long-Range Objectives for 2035.

[9] Wang T., Yin Z. (2023). Exploring Xi Jinping’s View of Marine Ecological Civilization from the Perspective of System Thinking. Mod. Commun..

[10] Yang H., Huo D., Xu Q. (2016). Views on modern marine ranching. Oceanol. Rt Limnol. Sin..

[11] Liu S., Zhou X., Zeng C., Frankstone T., Cao L. (2022). Characterizing the Development of Sea Ranching in China. Rev. Fish Biol. Fish.

[12] Ru X., Deng B., Feng Q., Lin C., Zhang L., Yang H. (2023). Comparison of Marine Ranching Constructions between China and Foreign Countries. J. Fish. China.

[13] Yang H.S. (2023). Current Advances and Prospects of Modern Fishery.

[14] She Y. (2008). Analysis on the development of marine ranches in South Korea and Japan and the necessity of carrying out this work in China. China Fish.

[15] Ling S. (2012). The enlightenment of the development experience of recreational fishery in the United States to the Yangtze River Delta. China Fish..

[16] Li B. (2012). A Study on China Marine Ranching Construction. Master’s Thesis.

[17] Ding D., Suo A. (2022). Theoretical thinking of artificial ecosystem for modern marine ranching. Bull. Chin. Acad. Sci..

[18] Loneragan N.R., Jenkins G.I., Taylor M.D. (2013). Marine stock enhancement, restocking, and sea ranching in australia: Future directions and a synthesis of two decades of research and development. Rev. Fish. Sci..

[19] Yang H., Ru X., Zhang L., Lin C. (2019). Industrial convergence of marine ranching and offshore wind power: Concept and prospect. Bull. Chin. Acad. Sci..

[20] Seitz R.D., Lipcius R.N., Knick K.E., Seebo M.S., Long W.C., Brylawski B.J., Smith A. (2008). Stock enhancement and carrying capacity of blue crab nursery habitats in Chesapeake Bay. Rev. Fish. Sci..

[21] Zhang X., Wang X., Tu Z., Zhang P., Wang Y., Gao T., Wang S. (2019). Current status and prospect of fisheries resource enhancement in Shandong Province. Chin. Fish. Econ..

[22] Taylor M.D., Brennan N.P., Lorenzen K., Leber K.M. (2013). Generalized predatory impact model: A numerical approach for assessing trophic limits to hatchery releases and controlling related ecological risks. Rev. Fish. Sci..

[23] Kitada S. (2018). Economic, Ecological and Genetic Impacts of Marine Stock Enhancement and Sea Ranching: A Systematic Review. Fish Fish..

[24] Xu X., Tang W., Wang Y. (2019). Releasing capacity of *Portunus trituberculatus* enhancement in Zhoushan fishing ground and Yangtze river estuary fishing ground and their adjacent waters. South China Fish. Sci..

[25] Tang Q. (1996). On the capacity and its research. Prog. Fish. Sci..

[26] Yang H., Zhang S., Zhang X., Chen P., Tian T., Zhang T. (2019). Strategic thinking on the construction of modern marine ranching in China. J. Fish. China.

[27] Zhang Y.Z., JIiang R.J., Liang J. (2020). The evaluation on conservation effect of fishery resources in marine ranching of Ma’an Archipelago area Zhejiang Shengsi. J. Zhejiang Ocean. Univ..

[28] Yang C., Wu Z., Liu H., Zhang P., Li W., Zeng X., Zhang X. (2016). The fishing strategy of *charybdis japonica* and *rapana venosa* and the carrying capacity of *Apostichopus japonucus* in Zhuwang, Laizhou artificial reef ecosystem based on Ecopath model. Period. Ocean. Univ. China.

[29] Sun L. (2011). Evaluation of Artificial Reef Construction in Shandong Province. Ph.D. Thesis.

[30] Xu Q., Zhang S. (2013). Site selection evaluation of marine ranching in Zhoushan area based on AHP method. J. Shanghai Ocean. Univ..

[31] Zhao X., Sun W., Ren G., Ma Y. (2014). Ecosystem health evaluation of Haizhou Bay marine ranching. Acta Laser Biol. Sin..

[32] Wang Y., Li Y., Xiao P., Shen S., Cai H., Song W. (2019). Evaluation of Social Effects of Artificial Reefs in the South Bay Islands by AHP. Ocean. Dev. Manag..

[33] Zhang R., Zhang Q., Zhao J., Wu Z., Liu H., Shou L., Liao Y.B., Liu Q., Tang Y., Zeng J. (2022). Using Ecopath Models to Explore Differences in Ecosystem Characteristics Between an Artificial Reef and a Nearby Natural Reef on the Coast of the North Yellow Sea, China. Front. Mar. Sci..

[34] Qin M., Wang X.R., Du Y.W., Wan X.L. (2021). Influencing factors of spatial variation of national marine ranching in China. Ocean. Coast. Manag..

[35] Wu Z.X., Zhang X.M., Lozano-Montes H.M., Loneragan N.R. (2016). Trophic flows, kelp culture and fisheries in the marine ecosystem of an artificial reef zone in the Yellow Sea. Estuar. Coast. Shelf Sci.

[36] Harrison S., Rousseau M. (2020). Comparison of artificial and natural reef productivity in Nantucket Sound, MA, USA. Estuaries Coasts.

[37] Wang X., Feng J., Lin C.G., Liu H., Chen M.Y., Zhang Y.L. (2022). Structural and functional improvements of coastal ecosystem based on artificial oyster reef construction in the Bohai Sea, China. Front. Mar. Sci..

[38] Yuan Y., Feng J., Xian W.W., Zhang H. (2022). Analysis of the ecosystem characteristics and ecological carrying capacity of the main commercial fish in the artificial reef ecosystem in Laizhou Bay using the Ecopath model. Sustainability.

[39] Li Z.P., Chen Y., Wang G., Mu J.D., Sun Y.F., Yu H.L., Xu J.L., Yan Y., Luo S.Y., Han F.Q. (2023). Ecological carrying capacity and carbon sequestration potential of bivalve shellfish in marine ranching: A case study in Bohai Bay, China. Front. Mar. Sci..

[40] Wootton J.T. (1998). Effects of disturbance on species diversity: A multitrophic perspective. Am. Nat..

[41] Chesson P. (2000). Mechanisms of maintenance of species diversity. Annu. Rev. Ecol. Syst..

[42] Ruiz G.M., Carlton J.T., Grosholz E.D., Hines A.H. (1997). Global invasions of marine and estuarine habitats by non-indigenous species: Mechanisms, extent, and consequences. Am. Zool..

[43] Zhang S., Zhou X., Wang K., Li J., Zhao J., Zhao X., Guo Y., Liu S., Cheng X. (2019). Review of marine livestock ecological urbanization hypothesis and marine ranching construction key-technology against blue growth background. J. Fish. China.

[44] Baird D., Ulanowicz R.E. (1989). The seasonal dynamics of the Chesapeake Bay ecosystem. Ecol. Monogr..

[45] Wulff J., Field K., Mann H. (1989). Coastal and Estuarine Studies. Coastal.

[46] Ulanowicz R.E. (1986). Growth and Development: Ecosystems Phenomenology.

[47] Ulanowicz R.E. (1997). Ecology: The Ascendant Perspective.

[48] Ulanowicz E.R. (2004). Quantitative methods for ecological network analysis. Comput. Biol. Chem..

[49] Libralato S. (2008). System Omnivory Index. Encyclopedia of Ecology.

[50] Biondi E., Casavecchia S., Pesaresi S., Zivkovic L. (2012). Natura 2000 and the Pan-European Ecological Network: A new methodology for data integration. Biodivers. Conserv..

[51] Heymans J.J., Coll M., Libralato S., Morissette L., Christensen V. (2014). Global patterns in ecological indicators of marine food webs: A modelling approach. PLoS ONE.

[52] Safi G., Giebels D., Larissa Arroyo N., Heymans J.J., Preciado I., Raoux A., Schueckel U., Tecchio S., De Jonge V.N., Niquil N. (2019). Vitamine ENA: A framework for the development of ecosystem-based indicators for decision makers. Ocean. Coast. Manag..

[53] De Jonge V., Kolkman M., Ruijgrok E., De Vries M. (2003). The need for new paradigms in integrated socio-economic and ecological coastal policy making. Proceedings of the 10th International Wadden Sea Symposium.

[54] De Jonge V.N., Pinto R., Turner R.K. (2012). Integrating ecological, economic and social aspects to generate useful management information under the EU Directives’ ‘ecosystem approach’. Ocean. Coast. Manag..

[55] Borrett S.R., Freeze M.A., Salas A.K. (2011). Equivalence of the realized input and output oriented indirect effects metrics in ecological network analysis. Ecol. Model..

[56] Wang D., Wu X., Ding L., Zhang M., Xie H., Zhang Q., Li S. (2022). A preliminary analysis of the ecosystem structure and function of the Yangtze Estuary based on Ecopath model. J. Environ. Eng. Technol..

[57] Yuan Y., Xian W., Zhang H. (2022). A review of the ecological carrying capacity of fishery resources in China based on the Ecopath model. Mar. Sci..

[58] Parsons T.R., Maita Y., Lalli C.M., Parsons T.R., Maita Y., Lalli C.M. (1984). Determination of chlorophylls and total carotenoids: Spectrophotometric method. A Manual of Chemical & Biological Methods for Seawater Analysis.

[59] (2007). Specifications for Oceanographic Survey—Part 6: Marine Biological Survey.

[60] Gulland J. (1965). Manual of methods for fish stock assessment. Part I: Fish populations analysis. FAO Fish. Tech. Pap..

[61] Willis T.J., Babcock R.C. (2000). A baited underwater video system for the determination of relative density of carnivorous reef fish. Mar. Freshw. Res..

[62] Christensen V., Walters C.J., Pauly D. (2005). Ecopath with Ecosim: A user’s guide. Fish. Cent. Univ. Br. Columbia Vanc..

[63] Winberg G.G. (1960). Rate of metabolism and food requirements of fishes. Belorussian State University, Minsk. Fish. Res. Board Can. Transl. Ser..

[64] Yang H.S. (2018). Monitoring and Biocapacity Evaluation of Marine Ranching Ecosystem.

[65] Link J.S. (2010). Adding rigor to ecological network models by evaluating a set of pre-balance diagnostics: A plea for PREBAL. Ecol. Model..

[66] Heymans J.J., Coll M., Link J.S., Mackinson S., Steenbeek J., Walters C., Christensen V. (2016). Best practice in Ecopath with Ecosim food-web models for ecosystem-based management. Ecol. Model..

[67] Chea R., Guo C.B., Grenouillet G., Lek S. (2016). Toward an ecological understanding of a flood-pulse system lake in a tropical ecosystem: Food web docstructure and ecosystem health. Ecol. Model..

[68] Ulanowicz R.E., Goerner S.J., Lietaer B., Gomez R. (2009). Quantifying sustainability: Resilience, efficiency and the return of information theory. Ecol Complex..

[69] Zeng Y., Zhao Y., Qi Z. (2021). Evaluating the ecological state of Chinese lake baiyangdian (BYD) based on ecological network analysis. Ecol. Indic..

[70] Tobor-Kapłon M., Holtkamp R., Scharler U., Doroszuk A., Kuenen F., Bloem J., Ruiter P. (2008). Evaluation of information indices as indicators of envrionmental stress in terrestial soils. Ecol. Model..

[71] Dong C., Zhou Y., Li J., Yang H., Yu Y., Shen L., Wu J. (2021). Water quality analysis of the Middle Yangtze River based on fuzzy comprehensive evaluation. Freshw. Fish..

[72] Wu X., Hu F. (2020). Analysis of ecological carrying capacity using a fuzzy comprehensive evaluation method. Ecol. Indic..

[73] U.S. GLOBEC (1996). Report on Climate Change and Carrying Capacity of the North Pacific Ecosystem.

[74] Odum E.P. (1968). Energy flow in ecosystems: A historical review. Am. Zool..

[75] Jiang W.M., Gibbs M.T. (2005). Predicting the carrying capacity of bivalve shellfish culture using a steady, linear food web model. Aquaculture.

[76] Cheung W.L., Watson R., Pitcher T. (2002). Policy simulation of fisheries in the Hong Kong marine ecosystem. The use of ecosystem models to investigate multispecies management strategies for capture fisheries. Fish. Cent. Res. Rep..

[77] Cheung W.-L. (2001). Changes in Hong Kong’s Capture Fisheries During the 20th Century and Reconstruction of the Marine Ecosystem of Local Inshore Waters in the1950s. Master’s Thesis.

[78] Christensen V., Walters C.J. (2004). Ecopath with Ecosim: Methods, capabilities and limitations. Ecol. Model..

[79] Harvey C.J. (2014). Mediation functions in Ecopath with Ecosim: Handle with care. Can. J. Fish. Aquat..

[80] Sadchatheeswaran S., Branch G.M., Shannon L.J., Moloney C.L., Robinson T.B. (2020). Modelling changes in trophic and structural impacts of alien ecosystem engineers on a rocky-shore island. Ecol. Model..

[81] Qiu C.G., Xu S.L., Qi C., Lin S.Z. (2013). Studies on early growth and development in *Sebastiscus marmoratus* with artificial breeding technology. J. Ningbo Univ..

[82] Yang C.H., Liu Q., Wang Y.B. (2022). Assessment of the maximum sustainable yield of *Portunus Trituberculatus* in the northern areas of the East China Sea under the impact of stock enhancement. Oceanol. Limnol. Sin..

[83] Yang H. (2016). Construction of marine ranching in China: Reviews and prospects. J. Fish. China.

[84] Boaventura D., Moura A., Leitao F., Carvalho S., Cúrdia J., Pereira P., de Fonseca L.C., dos Santos M.N., Monteiro C.C. (2006). Macrobenthic colonisation of artificial reefs on the southern coast of Portugal (Ancao, Algarve). Hydrobiologia.

[85] Walker S.J., Schlacher T.A., Schlacher-Hoenlinger M.A. (2007). Spatial heterogeneity of epibenthos on artificial reefs: Fouling communities in the early stages of colonization on an East Australian shipwreck. Mar. Ecol. Prog. Ser..

[86] Beck M., Brumbaugh R., Airoldi L., Carranza A., Coen L., Crawford C., Defeo O., Edgar G., Hancock B., Kay M. (2009). Shellfish Reefs at Risk: A Global Analysis of Problems and Solution.

[87] Cresson P., Ruitton S., Harmelin-Vivien M. (2014). Artificial reefs do increase secondary biomass production: Mechanisms evidenced by stable isotopes. Mar. Ecol. Prog. Ser..

[88] Liu Y., Zhao Y.P., Dong G.H., Guan C.T., Cui Y., Xu T.J. (2013). A study of the flow field characteristics around star-shaped artificial reefs. J. Fluids Struct..

[89] Zheng Y., Kuang C., Zhang J., Gu J., Chen K., Liu X. (2022). Current and turbulence characteristics of perforated box-type artificial reefs in a constant water depth. Ocean. Eng..

[90] Tong F., Chen G., Feng X., Liu Y., Chen P. (2022). The effect of the artificial reef on the structure and function of sediment bacterial community. Sustainability.

[91] Falcão M., Santos M., Vicente M., Monteiro C. (2007). Biogeochemical processes and nutrient cycling within an artificial reef off Southern Portugal. Mar. Environ. Res..

[92] Littler M.M., Littler D.S., Brooks B.L. (2010). The effects of nitrogen and phosphorus enrichment on algal community development: Artificial mini-reefs on the Belize Barrier Reef sedimentary lagoon. Harmful Algae.

[93] Lee M.O., Otake S., Kim J.K. (2018). Transition of artificial reefs (ARs) research and its prospects. Ocean. Coast. Manag..

[94] Li J., Zheng Y.-X., Gong P.-H., Guan C.-T. (2017). Numerical simulation and PIV experimental study of the effect of flow fields around tube artificial reefs. Ocean. Eng..

[95] Bortone S.A., Martin T., Bundrick C.M. (1994). Factors affecting fish assemblage development on a modular artificial reef in a northern Gulf of Mexico estuary. Bull. Mar. Sci..

[96] Addis D.T., Patterson W.F., Dance M.A., Ingram G.W. (2013). Implications of reef fish movement from unreported artificial reef sites in the northern Gulf of Mexico. Fish. Res..

[97] Grabowski J.H., Brumbaugh R.D., Conrad R.F., Keeler A.G., Opaluch J.J., Peterson C.H., Piehler M.F., Powers S.P., Smyth A.R. (2012). Economic Valuation of Ecosystem Services Provided by Oyster Reefs. Bioscience.

[98] Lima J.S., Atalah J., Sanchez-Jerez P., Zalmon I.R. (2020). Evaluating the performance and management of artificial reefs using artificial reef multimetric index (ARMI). Ocean. Coast. Manag..

[99] Christensen V., Pauly D., Christensen V., Pauly D. (1993). Improved Construction, Parametrization and Interpretation of Steady-State Ecosystem Models. Trophic Models of Aquatic Ecosystems.

[100] Lindeman R.L. (1942). The trophic-dynamic aspect of ecology. Ecology.

[101] Eddy T.D., Bernhardt J.R., Blanchard J.L., Cheung W.W., Colléter M., Du Pontavice H., Fulton E.A., Gascuel D., Kearney K.A., Petrik C.M. (2021). Energy flow through marine ecosystems: Confronting transfer efficiency. Trends Ecol. Evol..

[102] de la Chesnais T., Fulton E.A., Tracey S.R., Pecl G.T. (2019). The ecological role of cephalopods and their representation in ecosystem models. Rev. Fish Biol. Fish..

[103] Spitz J., Ridoux V., Trites A., Laran S., Authier M. (2018). Prey consumption by cetaceans reveals the importance of energy-rich food webs in the Bay of Biscay. Prog. Oceanogr..

[104] Connell J.H., Slatyer R.O. (1977). Slatyer mechanisms of succession in natural communities and their role in community stability and organization. Am. Nat..

[105] Walker L.R., Chapin F.S. (1987). Interactions among processes controlling successional change. Oikos.

[106] Pickett S.T.A., White P.S., Courtney S.P. (1985). The ecology of natural disturbance and patch dynamics. Science.

[107] Wu Z.L. (2020). Influence of Tropical Cyclones on Important Habitat of Marine Ranching in Shengsi Zhejiang Province: Take Typical Tropical Cyclone Events for Example. Ph.D. Thesis.

[108] Mccann K., Hastings A. (1997). Re-evaluating the omnivory–stability relationship in food webs. Proc. R. Soc. B Biol. Sci..

[109] Odum E.P. (1985). Trends expected in stressed ecosystems. BioScience.

[110] Scharler U.M., Baird D. (2005). A Comparison of selected ecosystem attributes of three south african estuaries with different freshwater inflow regimes, using network analysis. J. Mar. Syst..

[111] Jin X.S., Dou S.Z., Shan X.J., Wang Z.Y., Wang R.J., Bian X.D. (2015). Hot spots of frontiers in the research of sustainable yield of Chinese inshore fishery. Prog. Fish. Sci..

[112] Pauly D., Christensen V., Dalsgaard J., Froese R., Torres F. (1998). Fishing down marine food webs. Science.

[113] Liang C., Pauly D. (2017). Fisheries impacts on China’s coastal ecosystems: Unmasking a pervasive ’fishing down’ effect. PLoS ONE.

[114] Liang C., Pauly D. (2020). Masking and unmasking fishing down effects: The Bohai Sea (China) as a case study. Ocean. Coast. Manag..

[115] Chen Z.Z., Qiu Y.S., Jia X.P. (2008). Structure and function of Beibu Gulf ecosystem based on Ecopath model. J. Fish. Sci. China.

[116] Bohnsack J.A., Sutherland D.L. (1985). Artificial reef research: A review with recommendations for future priorities. Bull. Mar. Sci..

[117] Nakamura M. (1985). Evolution of artificial fishing reef concepts in Japan. Bull. Mar. Sci..

[118] Liang Z., Guo Z., Jiang Z., Zhu L., Sun L. (2020). Construction concept and technology of the marine ranching mode of the whole life history of fishes. J. Fish. China.

[119] Yang H., Ding D. (2022). Marine ranching version 3.0: History, status and prospects. Bull. Chin. Acad. Sci..

[120] Odum E.P. (1969). The Strategy of Ecosystem Development. Science.

[121] Lassalle G., Lobry J., Loc’H F.L., Bustamante P., Certain G., Delmas D., Dupuy C., Hily C., Labry C., Pape O.L. (2011). Lower Trophic levels and detrital biomass control the bay of Biscay Continental Shelf food web: Implications for ecosystem management. Prog. Oceanogr..

[122] Rooney N., Mccann K.S. (2012). Integrating food web diversity, structure and stability. Trends Ecol. Evol..

[123] Zhang X., Ji Q., Hu C., Xu H., Wang Y., Yang X., Guo H. (2023). Ecosystem stability of marine ranching and its response to disturbance: Research status, issues, and suggestions. J. Fish. China.

[124] Laikre L., Schwartz M.K., Waples R.S., Ryman N. (2010). Compromising genetic diversity in the wild: Unmonitored large-scale release of plants and animals. Trends Ecol. Evol..

[125] Lorenzen K., Beveridge M.C.M., Mangel M. (2012). Cultured fish: Integrative biology and management of domestication and interactions with wild Fish. Biol. Rev..

[126] Craig J.K., Link J.S. (2023). It is past time to use ecosystem models tactically to support ecosystem-based fisheries management: Case studies using Ecopath with Ecosim in an operational management context. Fish Fish..

[127] Shui B.N. (2023). Rethinking and optimization of releasing stock enhancement for marine fishery resources: A review. J. Dalian Ocean. Univ..

[128] Radinger J., Matern S., Klefoth T., Wolter C., Feldhege F., Monk C.T., Arlinghaus R. (2023). Ecosystem-based management outperforms species-focused stocking for enhancing fish populations. Science.

[129] Yang W., Zhang Z., Wu Y., Sun T., Liu H., Zhao Y. (2023). Ecosystem-based fishery management that accounts for trade-offs between fishing and enhancement stocking: A case study of Juehua island in the Bohai Sea. Ecol. Indic..

[130] Svåsand T., Kristiansen T.S., Pedersen T., Salvanes A.V., Engelsen R., Naevdal G., Nødtvedt M. (2000). The enhancement of cod stocks. Fish Fish..

[131] Bartley D.M., Bondad-Reantaso M.G., Subasinghe R.P. (2006). A risk analysis framework for aquatic animal health management in marine stock enhancement programmes. Fish. Res..

[132] Fordham D.A., Mellin C., Russell B.D., Akçakaya R.H., Bradshaw C.J., Aiello-Lammens M.E., Caley J.M., Connell S.D., Mayfield S., Shepherd S.A. (2013). Population dynamics can be more important than physiological limits for determining range shifts under climate change. Glob. Change Biol..

[133] Terborgh J.W. (2015). Toward a trophic theory of species diversity. Proc. Natl. Acad. Sci. USA.

[134] Cahoon L.B. (2002). The role of benthic microalgae in neritic ecosystems. Oceanography and Marine Biology.

[135] Hong X.F., Chen Z.Z., Zhang J., Jiang Y.E., Gong Y.Y., Cai Y.C., Yang Y.T. (2022). Analysis of ecological carrying capacity of reef organisms in Qilianyu Islands based on Ecopath model. J. Trop. Oceanogr..

[136] Holling C.S. (1973). Resilience and stability of ecological systems. Annu. Rev. Ecol. Syst..

[137] May R.M. (1977). Thresholds and breakpoints in ecosystems with a multiplicity of stable states. Nature.

[138] Sibly R.M., Hone J. (2002). Population growth rate and its determinants: An overview. Philosophical Transactions of the Royal Society of London. Ser. B Biol. Sci..

[139] Schaefer M.B. (1991). Some aspects of the dynamics of populations important to the management of the commercial marine fisheries. Bull. Math. Biol..

[140] Legović T., Perić G. (1984). Harvesting population in a periodic environment. Ecol. Model..

[141] Clark C.W. (1974). Mathematical bioeconomics. Mathematical Problems in Biology: Victoria Conference.

[142] Legović T., Klanjšček J., Geček S. (2009). The maximum sustainable yield leads to extinction of species in most single and multispecies fisheries. Nat. Preced..

[143] Inglis G., Hayden B.J., Ross A.H. (2000). An Overview of Factors Affecting the Carrying Capacity of Coastal Embayments for Mussel Culture.

[144] Filgueira R., Comeau L.A., Guyondet T., McKindsey C., Byron C. (2015). Modelling Carrying Capacity of Bivalve Aquaculture: A Review of Definitions and Methods.

[145] Hemraj D.A., Bishop M., Carstensen J., Krause-Jensen D., Stæhr P.A.U., Russell B.D. (2024). Nature protection must precede restoration. Science.

[146] Balbar A.C., Metaxas A. (2019). The current application of ecological connectivity in the design of marine protected areas. Glob. Ecol. Conserv..

[147] Ashley R., Russell D., Swallow B. (2006). The policy terrain in protected area landscapes: Challenges for agroforestry in integrated landscape conservation. Biodivers. Conserv..

[148] Yin J., Xue Y., Li Y., Zhang C., Xu B., Liu Y., Ren Y., Chen Y. (2023). Evaluating the efficacy of fisheries management strategies in china for achieving multiple objectives under climate change. Ocean Coast. Manag..

[149] Ahrens R.N.M., Walters C.J., Christensen V. (2012). Foraging arena theory. Fish Fish..

[150] Christensen V., Walters C.J., Pauly D., Forrest R. (2008). Ecopath with Ecosim Version 6 User Guide.

[151] Fath B.D., Asmus H., Asmus R., Baird D., Borrett S.R., de Jonge V.N., Ludovisi A., Niquil N., Scharler U.M., Schückel U. (2019). Ecological network analysis metrics: The need for an entire ecosystem approach in management and policy. Ocean. Coast. Manag..

[152] Christensen V. (1995). Ecosystem maturity towards quantification. Ecol. Model..

[153] Pauly D. (1980). On the interrelationships between natural mortality, growth parameters, and mean environmental temperature in 175 fish stocks. Ices J. Mar. Sci..

[154] Palomares M.L.D., Paulyb D. (1998). Predicting food consumption of fish populations as functions of mortality, food type, morphometrics, temperature and salinity. Mar. Freshw. Res..

[155] Lv X.M. (2016). Study on the Diet Composition of Juvenile of *Liza haematocheila* and *Konosirus punctatus* and Its Relationship with Ambient Phytoplankton. Master’s Thesis.

[156] Jiao Y., Chen D.G., Liu Q., Zhong C.J., Zeng X.Q., Ren Y.P. (2001). Biological characteristics of some small species in *Engraulidae* and *Clupeidae*. Trans. Oceanol. Limnol..

[157] Jiang R.J., Xue L.J., Zhang H.L., Zhu Z.J. (2013). Feeding habits of *Ilisha Elongata* in the East China Sea. Mar. Fish.

[158] Khan M.A., Yousuf K., Riaz S. (2014). Food and feeding habits of *Sillago Sihama* (Forsskal, 1775) (Family: *Sillaginidae*) from Karachi Coast. AkiNik Publ.

[159] Li Z.L., Zhang W.X., He X.B., Yan Y.R. (2019). Feeding ecology and feeding competition between *Decapterus maruadsi* and *Trachurus japonicus* in autumn in the Beibu Gulf, South China Sea. J. Guangdong Ocean. Univ..

[160] Yang G., Sun X., Hou X., Chen C. (2012). Measurement of the trophic level of fish in a coral reef ecosystem using stable isotopes. J. Fish. Sci. China.

[161] Zhang Y.M., Dai C.T., Yan Y.R., Yang Y.L., Lu H.S. (2014). Feeding habits and trophic level of crimson sea bream (*Parargyrops edita* Tanaka) in the Beibu Gulf. J. Fish. China.

[162] Jin H.W., Xue L.J., Zhu Z.J., Pan G.L. (2012). Feeding Habits of *Apogon Lineatusin* the East China Sea and Southern Yellow Sea. Mar. Fish.

[163] Liu J. (1997). Morphological comparison of the feeding apparatus of three co-occurring species of ponyfishes (*Leiognathidae*). Chin. J. Oceanol. Limnol..

[164] Yang L., Cao W.Q., Lin Y.S., Chen Y.H., Lin Z.J., Wang X.H. (2016). Preliminary study on feeding habits and trophic niche of nine economic fish species in Beibu Gulf in summer. J. Trop. Oceanogr..

[165] Yan Y.R. (2010). Feeding Ecology and Food Relations of the Main Fishes in the Beibu Gulf, South China Sea. Ph.D. Thesis.

[166] Wang K., Li C.W., Wang Z.H., Zhao J., Zhang S.Y. (2017). Feeding habits of the marbled rockfish *Sebastiscus Marmoratus* in the marine ranching off Ma’an Archipelago, China. Chin. J. Appl. Ecol..

[167] Han D., Xue Y., Ji Y., Xu B., Ma Q. (2013). Trophic and spatial niche of five gobiid fishes in Jiaozhou Bay. J. Fish. Ences China.

[168] Han D.Y. (2013). Study on Feeding Ecology of Dominate *Gobiidfishes* in Jiaozhou Bay. Master’s Thesis.

[169] Xu J.W., Zhang B., Zhang C.L., Xu B.D., Ji Y.P., Ren Y.P., Xue Y. (2022). Feeding ecology of *Amblychaeturichthys hexanema* in Haizhou bay based on linear mixed model. Chin. J. Appl. Ecol..

[170] Brey T. (1999). A Collection of empirical relations for use in ecological modelling. Naga ICLARM Q..

[171] Brey T. Population Dynamics in Benthic Invertebrates. A Virtual Handbook. Version 01.2, 2001. http://www.thomas-brey.de/science/virtualhandbook.

[172] Ning J., Du F., Wang X., Gu Y., Li Y. (2016). Feeding habits of mantis shrimp based on stable isotope analysis. J. Fish. China.

[173] Ning J.J., Du F.Y., Li Y.F. (2016). Dietary Composition and trophic position of blue swimmer crab (*Portunus Pelagicus*)in Honghai Bay. Haiyang Xue Bao.

[174] Huang M.Z. (2004). Study on feeding habits and food relationship of main economic invertebrates in Taiwan strait and its adjacent areas. Marine Sciences.

[175] Huang M.Z. (2004). Study on feeding habits and nutrient level of shrimp species from Taiwan Strait and its adjacent sea areas. J. Oceanogr. Tai Wan Strait.

[176] Yu J., Chen P., Feng X. (2016). Food habits and trophic levels for 4 species of economical shrimps in the Pearl River estuary shallow waters. J. South. Agric..

[177] Huang M.Z. (2004). Study on feeding habits and nutrient level of four cephalopod species from Taiwan Strait and its adjacent areas. J. Oceanogr. Tai Wan Strait.

[178] Mo B., Qin C., Chen P., Li X., Yuan H. (2017). Feeding habits of the purple sea urchin *Heliocidaris Crassispina* based on stable carbon and nitrogen isotope analysis. J. Fish. Sci. China.

[179] Ding M.M. (2019). Diet Assessment and Gut Microbiota of Intertidal Grazing Gastropods. Master’s Thesis.

[180] Lu J.P., Cai R.X., Qian Z.X. (1996). Stomach contents of the several barnacles in Zhoushan waters. Donghai Mar. Sci..

[181] Xu Q. (2007). Evaluation of Food Sources of Bivalve in Seaweed and Filter-Feeding Bivalve Polyculture Ecosystem. Ph.D. Thesis.

[182] Duan L.J., Li S.Y., Liu Y., Jiang T., Failler P. (2009). A Trophic model of the Pearl River Delta coastal ecosystem. Ocean. Coast. Manag..

[183] Wu Y.C. (2008). The Temporal and Spatial Patterns and Size-Fractioned Structure of Primary Productivity in Beibu Gulf. Ph.D. Thesis.

[184] Abdul W.O., Adekoya E.O. (2016). Preliminary Ecopath Model of a tropical coastal estuarine ecosystem around Bight of Benin, Nigeria. Environ. Biol. Fishes.

[185] Adebola T., Mutsert K.D. (2019). Spatial simulation of redistribution of fishing effort in Nigerian coastal waters Using Ecospace. Ecosphere.

[186] Akoglu E., Salihoglu B., Libralato S., Oguz T., Solidoro C. (2014). An indicator-based evaluation of black sea food web dynamics during 1960–2000. J. Mar. Syst..

[187] Arias-González J.E., Nuñez-Lara E., González-Salas C., Galzin R. (2004). Trophic models for investigation of fishing effect on coral reef ecosystems. Ecol. Model..

[188] Bella K., Sahadevan P., Raghavan R., Ramteke K.K., Sreekanth G.B. (2023). Trophic functioning of a small, anthropogenically disturbed, tropical estuary. Mar. Environ. Res..

[189] Bradford-Grieve J.M., Probert P.K., Nodder S.D., Thompson D., Hall J., Hanchet S., Boyd P., Zeldis J., Baker A.N., Best H.A. (2003). Pilot trophic model for subantarctic water over the southern plateau, New Zealand: A low biomass, high transfer efficiency system. J. Exp. Mar. Biol. Ecol..

[190] Campos W.L. (2003). An Ecosystem Model of San Pedro Bay, Leyte, Philippines: Initial Parameter Estimates. Work. Pap..

[191] Chen K., Meng Z., Li X., Hu F., Du H., Liu L., Zhu Y., Yang D. (2022). Community structure and functional diversity of fishes in Zhelin reservoir, Jiangxi Province. Acta Ecol. Sin..

[192] Chen X.P. (2023). A Study on the Community Structure and Trophic Dynamics of Fishery Resources in Dachen Island Area. Ph.D. Thesis.

[193] Chen Z., Qiu Y., Jia X. (2006). Mass-balance Ecopath model of Belbu Gulf ecosystem. Ying Yong Sheng Tai Xue Bao.

[194] Chen Z., Xu S., He P. (2011). An ecological model of the artificial ecosystem (Northern Hangzhou Bay, China): Analysis of ecosystem structure and fishing impacts. Helgol. Mar. Res..

[195] Chen Z., Xu S., Qiu Y. (2015). Using a food-web model to assess the trophic structure and energy flows in Daya Bay, China. Cont. Shelf Res..

[196] Coll M., Palomera I., Tudela S. (2009). Decadal Changes in a NW Mediterranean sea food web in relation to fishing exploitation. Ecol. Model..

[197] Coll M., Santojanni A., Palomera I., Tudela S., Arneri E. (2007). An ecological model of the northern and central Adriatic Sea: Analysis of ecosystem structure and fishing impacts. J. Mar. Syst..

[198] Colléter M., Gascuel D., Ecoutin J.M., Morais L.T.D. (2012). Modelling trophic flows in ecosystems to assess the efficiency of marine protected area (MPA), a case study on the coast of Senegal. Ecol. Model..

[199] Corrales X., Coll M., Tecchio S., Bellido J.M., Fernández A.M., Palomera I. (2015). Ecosystem structure and fishing impacts in the northwestern Mediterranean Sea using a food web model within a comparative approach. J. Mar. Syst..

[200] Dimarchopoulou D., Keramidas I., Tsagarakis K., Tsikliras A.C. (2019). Ecosystem models and effort simulations of an Untrawled Gulf in the central Aegean Sea. Front. Mar. Sci..

[201] Fourriére M., Alvarado J.J., Cortés J., Taylor M.H., Ayala-Bocos A., Azofeifa-Solano J.C., Arauz R., Heidemeyer M., López-Garro A., Zanella I. (2019). Energy flow structure and role of keystone groups in shallow water environments in Isla Del Coco, Costa Rica, Eastern Tropical Pacific. Ecol. Model.

[202] Garces L.R., Alias M., Abu Talib A., Mohamad-Norizam M., Silvestre G.T. (2003). A trophic model of the coastal fisheries ecosystem off the West Coast of Sabah and Sarawak, Malaysia, Assessment, management and future directions for coastal fisheries in asian countries. WorldFish Cent. Conf. Proc..

[203] Gurney L.J., Pakhomov E.A., Christensen V. (2014). An Ecosystem model of the Prince Edward Island Archipelago. Ecol. Model.

[204] Haggan N., Pitcher T. (2005). Ecosystem Simulation Models of Scotland’s West Coast and Sea Lochs. Univ. Br. Columbia Fish. Cent..

[205] Han R., Chen Q., Wang L., Tang X., Shen X. (2016). Analysis of the ecosystem structure and energy flow of the Yangtze River estuary and adjacent seas, based on the Ecopath model. Acta Ecol. Sin..

[206] Hernández-Padilla J.C., Zetina-Rejón M.J., Arreguín-Sánchez F., Del Monte-Luna P., Nieto-Navarro J.T., Salcido-Guevara L.A. (2021). Structure and function of the southeastern Gulf of California ecosystem during low and high sea surface temperature variability. Reg. Stud. Mar. Sci..

[207] Rybarczyk H., Elkaim B., Ochs L., Loquet N. (2003). Analysis of the trophic network of a macrotidal ecosystem: The Bay of Somme (Eastern Channel). Estuar. Coast. Shelf Sci..

[208] Heymans J.J., Baird D. (2000). Network Analysis of the Northern Benguela Ecosystem by means of NETWRK and ECOPATH. Ecol. Model..

[209] Hong J., He-Qin C., Hai-Gen X., Arrequín-Sánchez F., Zetina-Rejón M.J., Luna P.D.M., Le Quesne W.J. (2008). Trophic controls of jellyfish blooms and links with fisheries in the East China Sea. Ecol. Model.

[210] Zhao J., Zhang S.-Y., Xu M. (2010). The primary research of the energy flow in Gouqi kelp bed ecosystem. J. Shanghai Ocean. Univ..

[211] Li Y.K., Yu N., Chen L.Q., Chen Y., Feng D.X. (2010). Ecological modeling on structure and functioning of Southern East China Sea ecosystem. Prog. Fish. Sci..

[212] Lin H.J., Shao K.T., Kuo S.R., Hsieh H.L., Wong S.L., Chen I.M., Lo W.T., Hung J.J. (1999). A Trophic Model of a sandy barrier lagoon at Chiku in Southwestern Taiwan. Estuar. Coast. Shelf Sci..

[213] Lin Q., Jin X., Guo X., Zhang B. (2009). Study on the structure and energy flow of the Yangtze River estuary and adjacent waters ecosystem based on Ecopath Model. J. Hydroecology.

[214] Lin Q., Jin X., Zhang B., Guo X. (2009). Comparative study on the changes of the Bohai Sea ecosystem structure based on Ecopath model between ten years. Acta Ecol. Sin..

[215] Lin Q., Jin X.S., Zhang B. (2013). Trophic interactions, ecosystem structure and function in the southern Yellow Sea. Chin. J. Oceanol. Limnol..

[216] Lin Q., Li X.S., Li Z.Y., Jin X.S. (2013). Ecological Carrying Capacity of Chinese Shrimp stock enhancement in Laizhou Bay of East China based on Ecopath Model. Ying Yong Sheng Tai Xue Bao. J. Appl. Ecol..

[217] Lin Q., Shan X., Wang J., Li Z. (2018). Changes in Chinese Shrimp (*Fenneropenaeus chinensis*) carrying capacity of the Bohai Sea. Prog. Fish. Sci..

[218] Lin Q., Wang J., Li Z.Y., Wu Q. (2015). Assessment of ecosystem energy flow and carrying capacity of swimming crab enhancement in the Yellow River Estuary and adjacent waters. Chin. J. Appl. Ecol..

[219] Lira A., Angelini R., Le Loc’h F., Menard F., Lacerda C., Fredou T., Fredou F.L. (2018). Trophic flow structure of a neotropical estuary in northeastern Brazil and the comparison of ecosystem model indicators of estuaries. J. Mar. Syst..

[220] Liu H., Yang C., Zhang P., Li W., Zhang X. (2019). An Ecopath evaluation of system structure and function for the Laoshan Bay artificial reef zone ecosystem. Acta Ecol. Sin..

[221] Liu P.J., Shao K.T., Jan R.Q., Fan T.Y., Wong S.L., Hwang J.S., Chen J.P., Chen C.C., Lin H.J. (2009). A Trophic model of fringing coral reefs in Nanwan Bay, Southern Taiwan suggests overfishing. Mar. Environ. Res..

[222] Lozano-Montes H.M., Loneragan N.R., Babcock R.C., Jackson K. (2011). Using Trophic flows and ecosystem structure to model the effects of fishing in the Jurien Bay Marine Park, Temperate Western Australia. Mar. Freshw. Res..

[223] Ma M., Chen Z., Xu S., Zhang J., Yu W. (2020). Trophic structure and energy flow of continental slope of the northern South China Sea ecosystem. J. Fish. China.

[224] Ma M., Xu S., Xu Y., Zhang K., Yuan W., Chen Z. (2018). Comparative study of Jiaozhou Bay ecosystem based on an Ecopath model. J. Fish. Sci. China.

[225] Manickchand-Heileman S., Arreguín-Sánchez F., Lara-Domínguez A., Soto L.A. (1998). Energy flow and network analysis of Terminos Lagoon, SW Gulf of Mexico. J. Fish Biol..

[226] Monte-Luna P.D., Arreguín-Sánchez F., Lluch-Belda D. (2007). Marine ecosystem analyses in the gulf of Ulloa, Mexico: BAC meets Ecopath. NCOFISH ecosystem models: Transiting from Ecopath to ecospace. Fish. Cent. Res. Rep. Fish. Cent. Univ. Br. Columbia Vanc..

[227] Montero L.C., Christensen V., Hernández J.J.C. (2021). Simulating trophic impacts of fishing scenarios on two Oceanic Islands using Ecopath with Ecosim. Mar. Environ. Res..

[228] Morales-Zárate M.V., Lluch-Cota S.E., Serviere-Zaragoza E., del Próo S.G. (2011). Modeling an exploited rocky coastal ecosystem: Bahia Tortugas, Mexico. Ecol. Model..

[229] Okey T.A., Banks S., Born A.R., Bustamante R.H., Calvopiña M., Edgar G.J., Espinoza E., Fariña J.M., Garske L.E., Reck G.K. (2004). A trophic model of a Galapagos subtidal rocky reef for evaluating fisheries and conservation strategies. Ecol. Model..

[230] Ortiz M., Berrios F., Campos L., Uribe R., Ramirez A., Hermosillo-Núñez B., González J., Rodriguez-Zaragoza F. (2015). Mass balanced trophic models and short-term dynamical simulations for benthic ecological systems of Mejillones and Antofagasta bays (SE Pacific): Comparative network structure and assessment of human impacts. Ecol. Model..

[231] Ortiz M., Wolff M. (2002). Trophic models of four benthic communities in Tongoy Bay (Chile): Comparative analysis and preliminary assessment of management strategies. J. Exp. Mar. Biol. Ecol..

[232] Pinnegar J.K. (2000). Planktivorous Fishes: Links Between the Mediterranean Littoral and Pelagic.

[233] Rahman M.F., Qun L., Shan X.J., Chen Y.L., Ding X.S., Liu Q. (2019). Temporal changes of structure and functioning of the Bohai Sea ecosystem: Insights from Ecopath models. Thalass. Int. J. Mar. Sci..

[234] Ren X., Liu Y., Xu B., Zhang C., Ren Y., Cheng Y., Xue Y. (2020). Ecosystem structure in the Haizhou Bay and adjacent waters based on Ecopath model. Acta Oceanol. Sin..

[235] Rocha G.R.A., Rossi-Wongtschowski C.L.d.B., Pires-Vanin A.M.S., Soares L.S.H. (2007). Trophic models of São Sebastião channel and continental shelf systems, SE Brasil. Pan-Am. J. Aquat. Sci..

[236] Schückel U., Kröncke I., Baird D. (2015). Linking long-term changes in trophic structure and function of an intertidal macrobenthic system to eutrophication and climate change using ecological network analysis. Mar. Ecol. Prog. Ser..

[237] Steffen W., Sanderson A., Tyson P., Jäger J., Matson P., Moore B., Oldfield F., Richardson K., Schellnhuber H.J., Turner B. (2005). Reverberations of Change: The Responses of the Earth System to Human Activities. Global Change and the Earth System.

[238] Tecchio S., Rius A.T., Dauvin J.C., Lobry J., Lassalle G., Morin J., Bacq N., Cachera M., Chaalali A., Villanueva M.C. (2015). The mosaic of habitats of the Seine Estuary: Insights from food-web modelling and network analysis. Ecol. Model..

[239] Torres M., Coll M., Heymans J.J., Christensen V., Sobrino I. (2013). Food-web structure of and fishing impacts on the Gulf of Cadiz Ecosystem (South-Western Spain). Ecol. Model..

[240] Tsehaye I., Nagelkerke L.A.J. (2008). Exploring optimal fishing scenarios for the multispecies artisanal fisheries of eritrea using a trophic Model. Ecol. Model..

[241] Ullah M.H., Rashed-Un-Nabi M., Al-Mamun M.A. (2012). Trophic model of the coastal ecosystem of the Bay of Bengal using mass balance Ecopath Model. Ecol. Model..

[242] Wang T., Zhang H., Zhang H., Zhang S. (2016). Ecological carrying capacity of Chinese shrimp stock enhancement in Haizhou Bay of East China based on Ecopath model. J. Fish. Sci. China.

[243] Wang W., Wang J., Zuo P., Li Y., Zou X. (2019). Analysis of Structure and Energy Flow in Southwestern Yellow Sea Ecosystem Based on Ecopath Model. J. Appl. Oceanogr..

[244] Wang X.H., Li S.Y., Peng R.Y. (2009). Establishment and comparative analysis of Ecopath model of ecosystem evolvement in northern continental shelf of South China Sea. Mar. Environ. Sci..

[245] Wang Y., Gao L., Chen Y. (2018). Assessment of *Portunus* (*Portunus*) *Trituberculatus* (Miers, 1876) stock in the northern East China Sea. Indian J. Fish.

[246] Wolff M. (1994). A Trophic model for Tongoy Bay—A system exposed to suspended scallop culture (Northern Chile). J. Exp. Mar. Biol. Ecol..

[247] Xu C., Wang S., Zhao F., Song C., Zhuang P. (2018). Trophic structure and energy flow of the Yangtze estuary ecosystem based on the analysis with Ecopath model. Mar. Fish.

[248] Xu M., Yang X.Y., Song X.J., Xu K.D., Yang L.L. (2021). Seasonal analysis of artificial oyster reef ecosystems: Implications for sustainable fisheries management. Aquac. Int..

[249] Zhang S., Wang T., Fu X., Zhang H. (2015). A primary study on the energy flow in the ecosystem of fishery ecological restoration area in Haizhou Bay, Lianyungang. Mar. Environ. Sci..

[250] Zhang X., Xian W. (2016). Energy Flow and Network Analysis of the Yangtze Estuary ecosystem during 1985–1986. Mar. Sci..

[251] Zhang Y., Chen Y. (2007). Modeling and evaluating ecosystem in 1980s and 1990s for American lobster (*Homarus Americanus*) in the Gulf of Maine. Ecol. Model..

